# Genetic Diversity of Wheat Stripe Rust Fungus *Puccinia striiformis* f. sp. *tritici* in Yunnan, China

**DOI:** 10.3390/plants10081735

**Published:** 2021-08-23

**Authors:** Md. Ashraful Alam, Haoxing Li, Akbar Hossain, Mingju Li

**Affiliations:** 1 Yunnan Key Laboratory of Green Prevention and Control of Agricultural Transboundary Pests, Agricultural Environment and Resource Research Institute, Yunnan Academy of Agricultural Sciences, Kunming 650205, China; ashrafulw@yahoo.com (M.A.A.); haoxing.li@qq.com (H.L.); 2 Bangladesh Wheat and Maize Research Institute, Dinajpur 5200, Bangladesh; 3 College of Life Science and Technology, Huazhong Agricultural University, Wuhan 430070, China

**Keywords:** genetic diversity, population structure, wheat, *Puccinia striiformis* f. sp. *tritici*, Yunnan Province, China

## Abstract

The stripe rust of wheat is one of the devastating diseases in China, which is caused by fungus *Puccinia striiformis* f. sp. *tritici* (*Pst*). The Yunnan Province of China is located in the south-western part, and holds distinctive geographical and climate features, while wheat growth and epidemics of stripe rust fungus are fully dissimilar to the major wheat-growing regions of China. It is important to discover its origin and migration to control the disease. In this study, 352 isolates were sampled from 11 spots of the Yunnan Province during the wheat growing season from 2004 to 2015 and analyzed with SNPs markers of housekeeping genes. Results revealed that 220 haplotypes were inferred from the concatenating sequences; among them, 5 haplotypes (*viz*., ‘H86′, ‘H18′, ‘H8′, ‘H15′ and ‘H23′) comprised over 24.5% of the population. The haplotype diversity, nucleotide diversity, mutation rate and recombination events were 0.992, 6.04 × 10^−3^, 4.46 × 10^−3^ and 18.0 respectively, which revealed the genetic diversity of *Pst* populations among all locations. Four grouping methods, such as UPGMA-tree, PCA, PLS-DA and STRUCTURE, were employed for the categorization of the *Pst* populations conferring to their races and topographical localities. All methods were found significant and mostly had co-linear relations with each other. The analysis of molecular variance (AMOVA) conferred total variation was 9.09%, and 86.20% of variation was within the populations. The current study also exposed a comparatively high genetic multiplicity within the population, while low genetic inconsistency among the populations. Furthermore, the molecular records on the gene pole (*Nm* = 18.45) established that the migration of the stripe rust pathogen occurred among all locations in Yunnan province. The ancestral haplotype was detected in Yuxi. Based on the trajectories of upper airflow and genetic diversity of *Pst* populations in different locations, it is suggested that the locations Dehong, Dali, Lincang and Baoshan are probably a major source of *Pst* in Yunnan.

## 1. Introduction

Around the globe, the stripe rust disease of wheat is considered the most devastating disease, and it is caused by the fungus *Puccinia striiformis* f. sp. *tritici (Pst)* [1,2]. In terms of the area that can be affected by the disease, China is the largest epidemic region for stripe rust disease of wheat in the world [3]. During 1950, 1964, 1990 and 2002, yield losses of wheat due to the disease were ˃6.0, 3.0, 1.8 and 1.3 million metric tons, respectively [3,4]. Generally, stripe rust disease of wheat in China is the most devastating, due to distinctive inter-regional features that help to migrate the disease to a long distance over similar geographic regions [5]. It is noted that most of these races of *Pst* were first detected in the Gansu Province of China [6]. The genetic diversity of stripe rust pathogens was high in Gansu [6,7,8], where the *Pst* population can easily complete their lifecycle due to different elevations of mountains on wheat [9,10,11] and/or alternative hosts (*Berberis* spp.) for sexual reproduction [12,13].

The Yunnan Province is one of the wheat-producing Provinces in China, which is situated in the south-western part of the country [14]. From west to east, the Yunnan–Guizhou Plateau of China crosses this Province. This area shows an enormous dissimilarity of agro-climate, vegetation, agricultural systems as well as cultivation of wheat compared to other provinces in China. Wheat is generally cultivated in Yunnan province from the valleys’ lowland to highland, with overlapping growth stages of wheat along with the elevation, which delivers a year-round host for stripe rust pathogens [5,12]. A survey estimated that the incidence of numerous races of stripe rust fungus in Yunnan Province was dissimilar to that in original epidemic regions such as Gansu Province [5,6,7]. In addition, the earliest epidemic of stripe rust was recorded in Yunnan province of China in the 1930s [15]. Furthermore, Yunnan possesses this disease year-round, like Gansu [5].

Recently, in Europe, Australia and New Zealand, population structures of various pathogens have been recognized by using various molecular markers [15,16,17,18]. However, a recombination signature in *Pst* has been documented in Gansu Province [9,10]. In Europe, the genetic diversity of *Pst* fungus was exceptionally studied by Justesen et al. [19] by using the amplified fragment length polymorphism (AFLP) markers; similarly, in North America, Markell and Milus [20] also used the same markers for the identification of the genetic diversity of *Pst* populations. Single nucleotide polymorphisms (SNPs) are relatively different types of molecular markers and AFLP, RAPD (Random Amplification of Polymorphic DNA) and SSR (Simple Sequence Repeats) are replaced by SNPs with the development of sequencing technology. Polymorphisms can be detected in coding and non-coding regions by the SNP marker of an organism that can cover a large part of the genome [21]. A SNP is the mutation of a single base pair at a specific locus position, and SNPs can conserve during evolution [22]. In recent years, SNP markers have been used to study population structure in plant pathogens. Li et al. [5] exposed three SNP primers of housekeeping genes to study the origin, evolution and movement of *Pst* in China. Similarly, in the USA, Parks et al. [23] used SNP markers and found 25 haplotypes during the investigation of the population structure of *Blumeria graminis* f. sp. *tritici*. The current study aimed to investigate the genomic assortment and population structure of *Pst* in Yunnan Province from the year 2004 to 2015 by using SNPs markers of housekeeping genes.

## 2. Materials and Methods

### 2.1. Sampling and Multiplication

Wheat stripe rust samples were collected in the year of 2004 to 2015 (main wheat growing season from February to May) from eleven locations of Yunnan province in China. Naturally infected green leaves were taken randomly from different wheat nurseries and farmers’ fields (Table S1). The sampling distance between the two places was more than 1000 m. The sampling sites covered altitudes from 906 to 2480 m and the main profitable wheat cultivars to increase the multiplicity of the *Pst* pathogens. During sampling, a leaf of the wheat plant was collected as a sample and wrapped in a piece of clean paper. A total of 352 isolates were used in this study (Table 1).

Then, the dried sample leaf was placed on a wet filter paper in a Petri-dish, Φ100 mm, for 6 to 12 h in a dark place at a temperature of around 20 °C. The pustules were scraped with a dissecting needle and urediniospores spread to seedlings of ‘Mingxian 169′, grown in pots, Φ100 mm, when the first leaf was fully expanded. The inoculated seedlings were sprayed with water and kept in humid condition in a dark place for 24 h at a temperature of 10 ± 1 °C. The pots were then moved to a greenhouse covered with glass shade with an open-top to insulate each isolate. The temperature was kept at 14 ± 3 °C in the greenhouse with a lighting time of 10 to 14 h each day. Then, the fresh urediospores were collected with a test tube by tapping the tube when the symptoms were fully appeared and each pot was harvested 3 to 4 times to obtain about 10 mg of urediniospores. For DNA extraction, the spores of stripe rust pathogens were then shifted to a centrifugal tube, then desiccated and deposited/stored at 4 °C.

### 2.2. Primer Design

The sequences of *Pst* housekeeping genes were searched in Gen-Bank. Three protein-coding housekeeping genes were identified for developing SNP primers, namely heat shock protein 90 kDa (HSP), ubiquitin-activating enzyme E1 (UBA) and ubiquitin-conjugating enzyme E2 (UBC). The SNP primers were designed using Premier 5.0 software [https://en.freedownloadmanager.org/users-choice/Primer_Premier_5_64.html] (Accessed on 12 August 2021). Designed primer pairs were synthesized by Tsingke Biological Technology Co. Kunming, China. The other three primers were designed by Li et al. [22], namely, Elongation factor (EF-1), Map kinase 1 (MAPK) and Cyclin-dependent kinase 2 (CDC2). Details of all primers’ information are available in Table 2.

### 2.3. Procedures of DNA Extraction

The DNA was extracted directly from urediniospores by using a reformed cetyltrimethylammonium bromide (CTAB) technique, which was previously characterized by Chen et al. [24] with some modifications. For each segregate, 10 mg of urediniospores were taken into a 2.0 mL Eppendorf tube with 5–7 sterile glass balls (3–4 mm). Then, 500 μL of preheating abstraction buffer (50 mM Tris-HCl, pH 8.0, 150 mM NaCl and 100 mM EDTA) and 5 mL of protease (10 mg·mL^−1^) were added and shaken by vortex for 2 min, and the tube was then hatched at 65 °C for 60 min. The tube was cooled at room temperature, and 500 μL of chloroform was added, mixed gently and then centrifuged at 12,000 rpm for 10 min. Then, the supernatant was transferred into a 1.5 mL tube and 500 mL of pre-cooling isopropyl alcohol was added, mixed gently and kept for 30 min at −20 °C. After centrifuging for 10 min at 12,000 rpm, the discarded supernatant pellet was washed two times, then separately cold-washed with 70% ethanol and 100% ethanol, and then desiccated and melted in 40 μL of TE buffer. The DNA-solution was treated with RNase (final concentration 20 μg mL^−1^) and reserved for 60 min at 37 °C to entirely digest RNA. The DNA was re-hastened, rinsed with ethanol, dried and dissolved in 40 μL of TE buffer. The DNA concentrations were diluted to 20 ng μL^−1^ with TE buffer before storing at −20 °C in small aliquots.

### 2.4. PCR (Polymerase Chain Reaction) and Sequencing

The PCR was performed in a 20 μL volume, and all primers were amplified under similar warm air cycling circumstances and chemical reagent concentrations, except the annealing temperatures. Every reaction occupied 10 μL of TIANGEN 2 × Taq PCR Master-Mix (0.1 units of Taq Polymerase μL^−1^, 500 μM dNTPs each, 20 mM Tris-HCl (pH8.3), 100 mM KCl, 3 mM MgCl_2_ and other steadying and strengthening agents) 1 μL of each 10 μM primer, 1 μL of 20 ng of genomic DNA and 7 μL of ddH_2_O. The cycling situations were one cycle of 94 °C for 5 min, then 34 cycles of 94 °C for 25 s, strengthening (51–59 °C) for 25 s and 72 °C for 45 s, monitored by an ultimate extension phase of 72 °C for 5 min. Before sequencing, agarose gel electrophoresis used an output of 1 × TAE buffer for 40 min at 110 volts to perceive if the band is the distinctive directed band. Sequencing was carried out at Tsingke Biological Technology Co., Kunming, China. The sequencing instrument was a 3730 × l DNA Analyzer. The sequencing substance was Big-Dye Terminator v3.1.

### 2.5. Analysis of the Recorded Data

Recorded data were analyzed by the multi-evolutionary analysis software. The arrangements were aligned and split for a single gene and all of the samples by using MEGA 4.0 [25]. The sequences were concatenated conferring to the instruction of *Cdc2-*(*Ef-1α*)*-Hsp-Mapk1-Uba-Ubc*. The haplotypes, counting the records of SNP loci and the category, collapse sequence, ancestral and isolate number of haplotypes, were examined by Map tariff options for collapsing sequences and removing indels into haplotypes and eliminating infinite-sites’ desecrations, using SNAP Workbench 2.0 [26], and execution of numerous evolutionary analysis under a distinct interface [27]. It was assumed that all data were in accordance with the unrestricted model, and there was only a sole modification at an individually mutated locus. The diversity of haplotype (*Hd*) and nucleotide (*Pi*), neutrality tests (Tajima’s *D* and Fu’s *Fs* values), re-amalgamation incident (*Rm*) and coefficient of genetic differentiation (*Gst*) based on haplotypes and gene flow (*Nm*) was calculated in DnaSP v.5.10 [28]. Negative and significant *D* and *Fs* values were taken as one source of the indications of population expansion. The mutation rate of the populations (*Θ*) was computed in MEGA 4.0. Analysis of molecular variance (AMOVA) was carried out using Arlequin 3.1 [29]. AMOVA is a technique of segregating genetic assortment into within-population and between populations for distinguishing population dissimilarities [30]. To assess the degree of isolates’ concentration changes (heat maps), PCA and PLS-DA clustering were performed using the Metabo-Analyst 2.0 software [31]. Heatmaps were created based on the Pearson distance. The UPGMA-tree was constructed using MEGA 5.0 software [25] and illustrated by FigTree v1.4.2. The STRUCTURE 2.3.4 was used for inferring population structure [32]. For the Evanno plot, the Structure Harvester was followed for imagining the structure outputs [33].

Bayesian-based clustering was performed using STRUCTURE v.2.3.4 [32], testing three independent runs with K from 1 to 14, with each run having a burn-in period of 50,000 iterations and 500,000 Monte Carlo Markov iterations, assuming an admixture model. The most likely K value was processed with STRUCTURE HARVESTER v.0.9.94 [33] and was detected using the Evanno transformation method [34]. To assign samples to clusters, a membership coefficient of *q* > 0.8 was used, while coefficients ≤ 0.8 were considered “genetically admixed”.

## 3. Results

### 3.1. Genetic Diversity in the Yunnan Pst Isolates

From 11 counties of Yunnan province in the years of 2004 to 2015, 220 haplotypes were detected from 352 samples using 6 SNP primers collected. There were 42 SNP loci samples collected from all locations, where 33 were phylogenetically informative (Table S2). No supplements or removals were identified, and all recorded data were constant with an infinite-sites model, where each variable locus has only a distinct metamorphosis. By using 6 primers, a total of 1354 polymorphic alleles were found across all populations (Table S2). Among them, 161 polymorphic alleles were detected across SNP primer CDC2, 88 were detected across EF-1, 379 were detected across HSP, 218 were detected across MAPK-1, 147 were detected across UBA and 359 were detected across UBS (Supplementary Table S2). There were 25, 24, 19, 30, 23, 30, 31, 22, 22, 22 and 37 haplotypes found in Lijiang (LJ), Dehong (DH), Baoshan (BS), Dali (DL), Qujing (QJ), Zhaotong (ZT), Yuxi (YX), Lincang (LC), Wenshan (WS), Chuxiong (CX) and Kunming (KM), respectively (Table 3).

**Figure 1:**
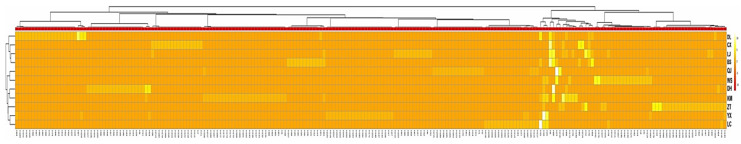
Heat-map visualization and hierarchical clustering analysis with Metabo-Analyst’s data annotation tools were constructed based on the different haplotypes for 11 locations. Rows: locations; Columns: haplotypes. Color key indicates haplotypes value, red: lowest, white: highest.

The private haplotypes were 13 in Lijiang (LJ), 18 in Dehong (DH), 10 in Baoshan (BS), 20 in Dali (DL), 16 in Qujing (QJ), 17 in Zhaotong (ZT), 21 in Yuxi (YX), 12 in Lincang (LC), 13 in Wenshan (WS), 15 in Chuxiong (CX) and 26 in Kunming (KM), respectively. Private haplotypes are those haplotypes that are found in one particular population sample but are absent in the samples from other populations. Haplotypes H86, H18, H8, H15 and H23 had the maximum incidence among the haplotypes, which added up to 24.5%, and were shared in the populations of Dehong, Yuxi, Lincang, Qujing and other counties (Figure 1; details in Table S3). Among them, H18 and H86 were comparatively widespread haplotypes and shared in six counties. The haplotypes were distributed at altitudes of 906 to 2480 m and were composed of local and introduced varieties and near-isogenic lines (Table S1). We constructed a dendrogram using Metabo-Analyst analysis to infer phylogenetic relationships among the *Pst* populations within the locations. The locations were assembled into six groups. Group 1: Qujing and Wenshan, Group 2: Zhaotong, Group 3: Yuxi and Lincang, Group 4: Chuxion, Lijiang and Baoshan, Group 5: Dali and Group 6: Dehong and Kunming (Figure 1 and Figure S1).

The outcomes of the diversity of haplotypes designated that the maximum *Hd* value was in Zhaotong, 0.993, and the lowest was in Qujing, 0.946 (Table 4). The diversity of nucleotides (*Pi*) fluctuated from 3.91 × 10^−3^ to 5.98 × 10^−3^ in the diversified populations. The maximum was in Wenshan and the lowest in Lijiang. The mutation rate was the highest in the Wenshan population (5.98 × 10^−3^) and lowest in the Baoshan (3.34 × 10^−3^) population. The recombination tests revealed that Zhaotong and Kunming had the maximum recombination, with *Rm* = 11, and it was lowest in Lijiang, *Rm* = 6 (Table 4).

The overall Tajima’s *D* was positive and not significant (*D* = 0.8238, *p* = 0.77364), indicating low levels with low-frequency polymorphisms within locations. The Fu’s *Fs* was highly significant and negative (*Fs* = −369.901, *p* = 0.0000), indicating an excess number of alleles, as would be expected from a recent population expansion or genetic hitchhiking. The individual Tajima’s *D* values for populations of different counties in Yunnan were positive and not significant, except Lijiang and Dehong. Lijiang and Dehong were negative and not significant (Table 4). Fu’s *Fs*, which was considered extra subtle to population demographic expansion [35], displayed different results. Fu’s *Fs* was undesirable and extremely substantial for all counties’ populations. The ancestral haplotype (H148) was detected in Yuxi, but all other results indicated frequent pathogen exchange within the locations (Table 4). The sequence of H148 is listed in Supplementary Table S2. The phylogeny tree (UPGMA) of haplotypes indicated that some haplotypes collected from the different locations were grouped, such as H4 from Wenshan, H6 from Lijiang and H161 from Kunming. Some haplotypes were from the same locations but grouped to different clusters, such as H1 and H2 from Qujing and H6 and H8 from Lijang. This indicates that the clustering of haplotypes was not related to geographical sources.

The coefficient of genetic differentiation (*Gst*) among all populations of Yunnan was 0.01337, while it was 0.0123, 0.00875, 0.01199, 0.00819, 0.02104, 0.00634, 0.00495, 0.00307, 0.0046 and 0.01126 between Yuxi and Lijiang (LJ), Dehong (DH), Baoshan (BS), Dali (DL), Qujing (QJ), Zhaotong (ZT), Kunming (KM), Lincang (LC), Wenshan (WS) and Chuxiong (CX), indicating a low differentiation among the ten counties, except Qujing (Table 5). The *Gst* was low among all populations, indicating lower heterogeneity.

Among all populations in Yunnan, the gene flow strength, *Nm,* was 18.45, indicating a recurrent pathogen interchange among the provinces (Table 5). While computing the tradeoff of *Pst* between Yuxi (YX) and Lijiang (LJ), Dehong (DH), Baoshan (BS), Dali (DL), Qujing (QJ), Zhaotong (ZT), Kunming, Lincang (LC), Wenshan (WS) and Chuxiong (CX) Provinces, the *Nm* was 20.08, 28.33, 20.61, 30.28, 11.63, 39.19, 50.30, 81.17, 54.09 and 21.96, respectively. *Gst* was the lowest, 0.00307, and *Nm* the highest, 81.17, between Yuxi and Lincang. These results indicated that *Pst* was extremely consistent in Yuxi and Lincang as compared to other provinces, and there was a huge scale of pathogen substitution between the two provinces. The results of AMOVA signposted that modification largely originated from within populations, accounting for 86.20% (Table 6), while it accounted for 9.09% among populations within assemblies and accounted for 4.71% among clusters.

**Figure 2:**
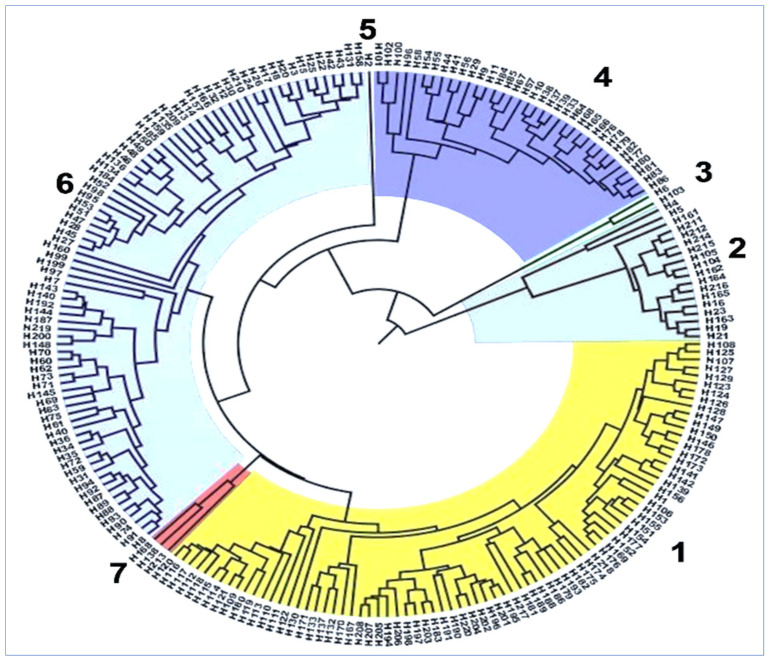
UPGMA dendrogram based on dissimilarity index of 42 SNP loci for 220 haplotypes.

The dendrogram (Figure 2) was prepared from the genetic variation matrix derivatives from 42 SNP loci for 220 haplotypes. In the UPGMA (unweighted pair-group method using arithmetic averages) dendrogram, the haplotypes were assembled into seven groups; however, three of them contained less than five haplotypes. An additional 4 groups were characterized as key groups comprising more than 15 haplotypes. Group 1 contained 79 haplotypes with 92 isolates, where 85.7% of isolates were from Yuxi (17), Kunming (14), Lincang (14), Wenshan (13), Chuxiong (12) and Qujing (8). Group 2 contained 18 haplotypes with 38 isolates, where 89% of isolates were from Qujing (16), Wenshan (6), Zhaotong (6) and Dali (4). Group 4 contained 35 haplotypes with 81 isolates, where 68% of isolates were from Dehong (22), Kunming (20) and Lijiang (12). Group 6 contained 82 haplotypes with 135 haplotypes, where 79% of isolates were from Dali (24), Baoshan (17), Yuxi (17), Zhaotong (13), Kunming (12) and Qujing (8).

**Figure 3:**
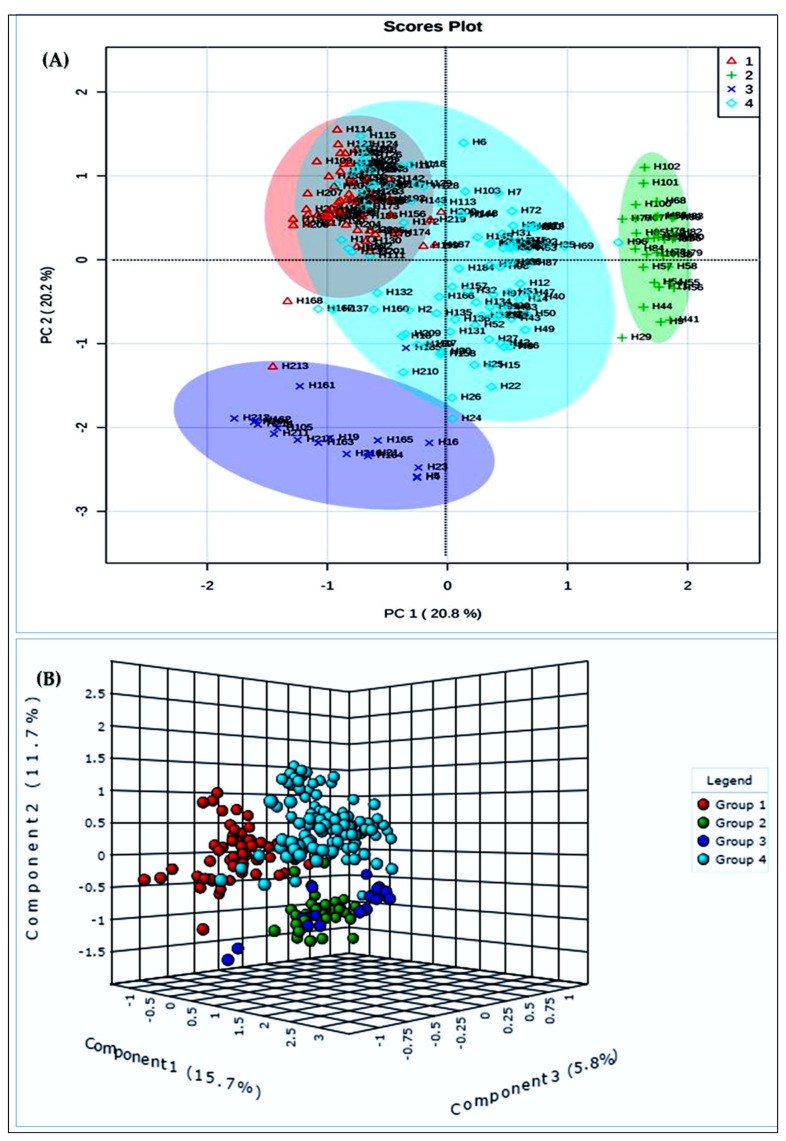
(**A**)**.** Principal component analysis of 220 haplotypes based on SNP markers’ data; (**B**) Partial least squares-discriminant analysis of 220 haplotypes based on SNP markers’ data.

Principal component analysis (PCA) was used as a way to deliver a three-dimensional graphical image of the proportional genetic detachments between the populations. It also measures the strength of the diversity between the groups categorized by a dendrogram. The haplotypes grouped by PCA and PLS-DA were carefully arranged with a UPGMA-based tree. In PCA scatterplots, the first two principal components explained 20.8% and 20.2% (Figure 3A), and in PLS-DA, the first three principal components explained 15.7%, 11.7% and 5.8% (Figure 3B) of the entire dissimilarity, respectively. In agreement with the UPGMA-tree, haplotypes were obviously detached by PC1 and 4 distinct groups were found; however, group 1 and group 4 were very close. In PLS-DA, the haplotypes were also found in 4 distinct groups, and group 2 and group 3 were close.

### 3.2. Population Structure of the Yunnan Pst Isolates

**Figure 4:**
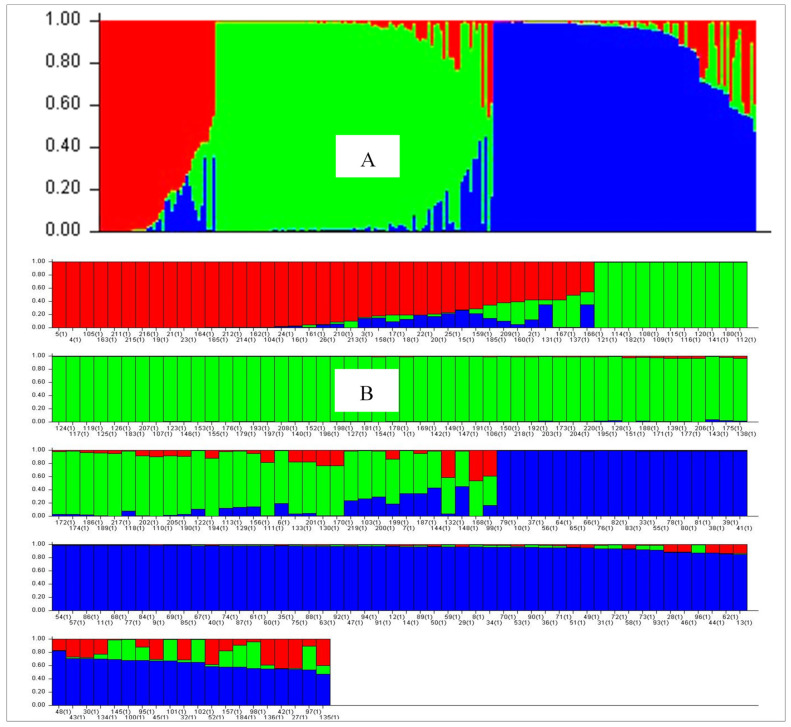
Model-based population structure plot for each isolate with K = 3, using structure with SNP markers’ data. Note: Color codes are as follows: Population I: red, Population II: green, Population III: blue. (**A**) The color code of each haplotype corresponds to the description in (**B**).

For population genetic structure analysis, Bayesian clustering modeling was performed in the STRUCTURE software using 220 haplotypes, where data were generated by SNP markers. As the clustering model assumes the fundamental reality of K clusters, an Evano test was carried out and generated K = 3 as the maximum log-likelihood (Figure S2; Figure 4).

This means that three was the optimal number of sub-populations, representing that all populations characterize three dissimilar clusters. The analysis of structure according to the geographical origin was performed by setting the range of a possible number of sub-populations (K) from 2 to 10. In STRUCTURE software analysis, concurrences were further characterized as unadulterated or admixture, where concurrences with a score > 0.80 were measured as pure and < 0.80 as an admixture. The population I comprised 17.7% of haplotypes (39 haplotypes), where 24 haplotypes were pure and 15 were admixed. There was a total of 90 isolates in population I, with 20 isolates from Qujing, 10 from Zhaotong, 9 from Lincang, 9 from Kunming, 8 from Dali and 9 from Wenshan, which covered 72% of the population I isolates. Population II comprised of a total of 93 haplotypes with 115 isolates, of which, 12 haplotypes were found admixed. Out of 113 isolates, 25 isolates from Yuxi, 20 isolates from Kunming, 13 isolates from Chuxiong, 12 isolates from Wenshan, 15 isolates from Lincang, 10 isolates from Zhaotong and 9 isolates from Qujing covered 92% of population II isolates. In population III, there was a total of 88 haplotypes with 149 isolates, where 19 were admixed. Out of 149 isolates, 5 locations, Dehong (25), Dali (25), Lijiang (19), Baoshan (19) and Kunming (19), covered 71.8% of the population III isolates (Figure 4). The incidental descent particulars with SNP markers for the strength of character of population structure of the 220 haplotypes are specified in Table S2.

## 4. Discussion

### 4.1. Genetic Diversity and Population Structure

The results from our study demonstrated that the genetic diversity, as well as the mutation rate of the Pst population, is very high in different locations of Yunnan. The strong ultraviolet rays due to the high altitude may be the drivers of mutation. Mutation is the important process for virulence variation [5]. The climate and the agricultural practices provide the year-round growth of wheat or volunteer wheat plants to ensure the emergence of new mutants in Yunnan. Previously, several population structure studies of *Pst* were conducted in China. Zeng and Luo [7] classified 15 stripe rust epidemiological regions in China. Wan et al. [3] and Chen et al. [35] recognized that the southern part of Gansu Province was the main source of *Pst* in China. Hu et al. [36] revealed 13 natural populations in Gansu, Shaanxi, Sichuan and Tibet, and the genetic diversity was highest in Gansu and Sichuan populations. Recently, Ali et al. [37] mentioned that the Himalayas region, such as China, seems more likely the center of origin for stripe rust pathogens worldwide, recognized based on the presence of the maximum levels of multiplicity, secluded alleles, the structure of the recombinant population, the capability to produce sex-related structures and the self-governing preservation of *Pst* pathogens’ center. Li et al. [5] conducted a study in Yunnan, Guizhou, Sichuan and Shaanxi, analyzing their data combined with the trajectory analysis of upper airflow, and presented to the overall story of the pathogen migration and revised that Yunnan is the main source of *Pst* in China. Wang et al. [38] documented that the genetic diversity was reliably high in Gansu and Shaanxi, but low in Sichuan, and there was a closer relationship between Gansu and Sichuan. A total of 1454 multi-locus genotypes (MLGs) were detected in the USA from 2010 to 2017 and observed that populations in the western part were more MLGs and higher divergence than in the eastern part of country [39].

Recombination of *Pst* was first reported in Yunnan by Li et al. [5]. Our study has intensively investigated different counties of Yunnan province. To explore the molecular genetic variation of the wheat stripe rust population, we adopted SNP neutral markers to carry out multi-locus sequence typing analysis on the *Pst* population. The phylogenetic investigation of *Pst* exhibited a structure in which long-distance dispersion and self-regulating progression harmonize. The haplotype diversity of the pathogen population was high (*Hd* = 0.992), and the pathogen diversity was rich in different counties in Yunnan. This is probably due to the diverse geographic environment and complex climate, and the natural conditions differ extremely between regions. It is, therefore, likely that the gene-flow is close to an island model between Yuxi and Lincang, and it is also more like a neighborhood model between Yuxi and Lincang (Table 5 and Figure 1).

Of these 220 detected haplotypes, H18 and H86 had a high frequency, occurred in the 6 areas and represented a stable genotype that was the best adapted to the current environment in the history of pathogen evolution. Other haplotypes that had lower frequencies probably occurred more recently in history or had lower adaptation. The high gene-flow of the pathogen population (*Nm* = 18.45) suggested a frequent exchange between the sub-populations. Considering all locations, the gene-flow was positive and significant, suggesting a higher exchange of *Pst* isolates within locations. The evolution analysis (*D* = 0.8238, *p* = 0.77364 and *Fs* = −369.901, *p* = 0.0000) of population structures suggested that there was a leftover digit of alleles, as would be anticipated from a current population extension or from genomic hitching in the Yunnan population. This is in accordance with the relatively low divergence among populations (9.09% variation). This provides the DNA proof for the long-distance dispersal of stripe rust pathogens [5,32,34] and the possible explanation as to why wheat stripe rust affects Yunnan Province year-round. The high level of genetic diversity also indicated a rapid population growth after a bottleneck [38,40]. This agrees with the results of surveys carried out in the past years [5]. In previous studies, the ancestral haplotype was detected in Yunnan, indicating that the pathogen of *Pst* in Yunnan was older [5]. It was also suggested that Yunnan is the center of origin of *Pst* in China. Using AFLP, the population of *P. striiformis* in Yunnan Province has been detected as a clonal population [41].

There was no significant difference in the diversity of haplotypes and nucleotides within all populations of Yunnan (Table 4). All regions likely play a significant role in supplying new emergences. The mutation rate of the pathogen was the highest (*θ* = 4.46 × 10^−3^) considering all tested populations (locations) in Yunnan. In accordance with the mutation rate, the recombination events were high (*Rm* = 18), suggesting that the new pathotype emerged in Yunnan due to mutation. The virulence metamorphosis is a significant process of virulence variation [38]. Li et al. [5] also observed the high mutation rate (*θ* = 3.81 × 10^−3^) and recombination event (*Rm* = 5) in Yunnan in 2008 and 2011, indicating that the new pathotype emerged earlier in Yunnan. As mentioned above, the strong ultraviolet rays due to the high altitude may be the drivers of mutation. The topography, the elevation between 77 and 6749 m, the climate and the farming practices provide the year-round growth of wheat or volunteer wheat plants and ensure the emergence of new mutations and variations in Yunnan [5]. The element factors related to the formation of such dynamic genetic structure may include mutation, recombination, host selection, the size and composition of an incurred population, the distance the wind can reach, the hitch-hiking, if there are alternate hosts or not, etc.

The expansion of the *Pst* population was measured by Tajima’s *D* tests in different locations in Yunnan, suggesting that the *Pst* population acts as a source of dispersal. Among all populations in Yunnan, the gene-flow strength, *Nm,* was 18.45, indicating a recurrent pathogen interchange among the provinces (Table 5). Li et al. [5] documented that the *Nm* was extensively higher in different locations of Yunnan: 142.60 and 9.47 for the two years studied (2008 and 2011). Chen [40] studied 20 natural populations of *Pst* in the main epidemiological region and found that the *Nm* values of Shaanxi and Gansu ranged from 1.1 to 9.0, and the highest *Nm* values between Gansu and Sichuan varied from 1.1 to 2.5. Hu et al. [36] stated that the populations of Gansu, Shaanxi and Sichuan of China had extensive gene exchange (*Nm* > 4) compared to Tibet. *Gst* was the lowest, 0.00307, and *Nm* the highest, 81.17, between Yuxi and Lincang. These results indicated that *Pst* was extremely consistent in Yuxi and Lincang as compared to other provinces and there was a huge scale of pathogen substitution between the two provinces.

The UPGMA-trees formed by SNP markers were similar to the outcomes of structure. Most of the isolates from groups 2 and 5 were allocated to population I of the structure. Groups 1, 3 and 7 were allocated to population II, and most of the isolates from group 4 were allocated to population III. The isolates from group 6 were distributed among three population groups. Population structure analysis showed that 8% of haplotypes were highly admixed. Using the SNP dataset, most of the genomic diversity has been clarified by the first axis of the PCA investigation. Nevertheless, SNP indicators were established as insignificant for the group of the existing set of segregates to some magnitude, rendering to their topographical positions. All four methods (UPGMA-tree, PCA, PLS-DA and STRUCTURE) were applied in the present study to categorize the *Pst* populations conferring to their races and physical sites, which were recognized as significant, and the furthermost of clutches were co-linear in all methods [6].

The present study also aimed to estimate the genetic relationship among populations of stripe rust pathogens in different locations of Yunnan province. AMOVA based on multi-locus sequences revealed a lower genetic differentiation among populations (9.09%), and most of the diversity was due to individuals within the populations (86.20%), indicating that the genetic divergence of the pathogen mainly came from inside the population. All these results indicate that the *Pst* population changes quickly. A lower level of genomic assortment between populations and a higher level within the population in Yunnan Province [5,38]. It is reported that there was geographic divergence for both wheat and stripe rust [5]. Bai et al. [39] also stated that the genetic variation was higher among years in the USA using AMOVA.

### 4.2. Route of Pst Dispersal in Yunnan

**Figure 5:**
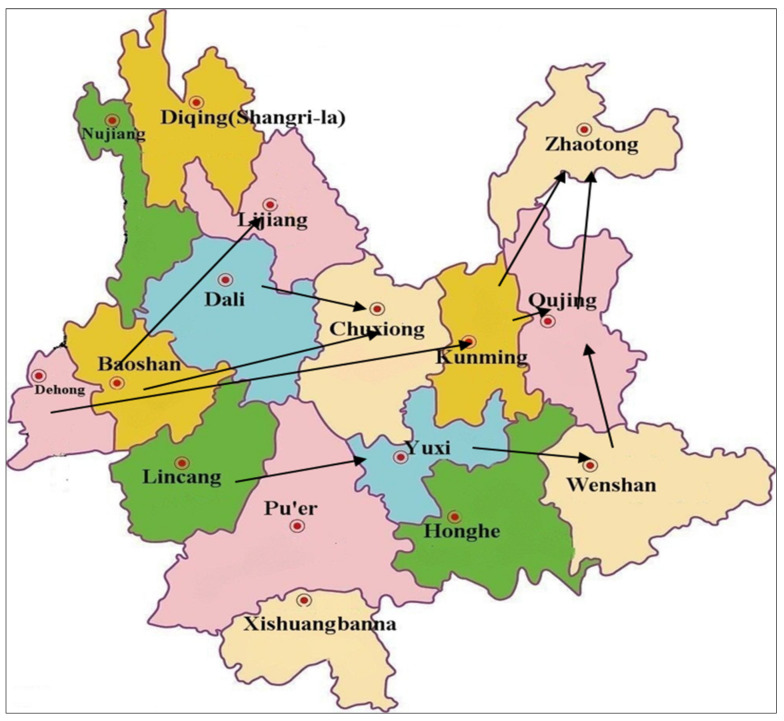
Route of stripe rust pathogen dispersal in Yunnan.

China is constantly under the westerly winds, the Himalayas are located at the border between China and countries west of China, and the wind that may carry urediniospores blows, along the south face of the Himalayas, into southwestern China, e.g., Yunnan, from the countries such as Pakistan, Nepal, etc. [37]. Then, *Pst* evolves locally and independently and disperses further to the northeast and the northern part of China. Yunnan, having all the characteristics of being a center of origin, provides new incursions and new emergences to the northern regions of Yunnan, including Gansu. Li et al. [5] suggested that Yunnan is the primary source of *Pst* in china. Our results also suggested that the high genetic diversity of *Pst* isolates is present in different counties of Yunnan. The exchange rate was also high within the populations. The trajectory of upper airflow is the main indicator for the detection of *Pst* urediniospores’ dispersal. The earlier studies performed by Li et al. [5], looking at the trajectories of upper airflow between Yunnan and Gansu during wheat growing seasons from 2005 to 2012, showed that the direction of airflow was from southwest Yunnan to north and northeast Yunnan. In our study, we intensively analyzed the population structure in different locations of Yunnan. Based on the trajectories of upper airflow and genetic diversity (Figure 1) of *Pst* populations in different locations, we suggested that Dehong, Dali, Lincang and Baoshan are probably the sources of *Pst* in Yunnan (Figure S3; Figure 5). 

## 5. Conclusions

In this study, results from SNPs of 352 segregates showed that 6 housekeeping genes were established to comprise a total of 42 SNP positions. From the concatenated sequences, 220 haplotypes were found, with 5 haplotypes (*viz*., ‘H86′, ‘H18′, ‘H8′, ‘H15′ and ‘H23′) comprising over 24.5% of the population. The haplotype diversity, nucleotide diversity, mutation rate and recombination events were 0.992, 6.04 × 10^−3^, 4.46 × 10^−3^ and 18.0 respectively, which revealed the genetic diversity of *Pst* populations among all locations. Four grouping methods, UPGMA-tree, PCA, PLS-DA and STRUCTURE, were applied in the present study to categorize the *Pst* populations, conferring to their races and physical localities, and the majority of the groups were co-linked in all methods for grouping. By using AMOVA, the study recognized about 9.09% of total dissimilarity, and 86.20% within populations. The findings of the study also showed that comparatively, the maximum hereditary assortment resulted from inside the population, but lower genetic discrepancy was found among populations. Furthermore, the genomic data on gene-flow (*Nm* = 18.45) established that the movement of pathogens occurred among all locations in Yunnan Province. Based on the trajectories of upper airflow and genetic diversity of *Pst* populations in different locations, it is suggested that Dehong, Dali, Lincang and Baoshan are probably the sources of *Pst* in Yunnan.

## Figures and Tables

**Table 1:** The numbers of *Puccinia striiformis* f. sp. *tritici* isolates were obtained from 11 different locations of Yunnan from 2004 to 2015 (main wheat growing season: February to May).

Location	Year						Total
	2004	2008	2011	2012	2014	2015	
Lijiang (LJ)	16	-	12	4	-	-	32
Dehong (DH)	-	3	19	-	2	5	29
Baoshan (BS)	-	16	7	-	-	-	23
Dali (DL)	-	13	13	-	10	-	36
Qujing (QJ)	-	22	-	-	5	5	32
Zhaotong (ZT)	5	13	4	-	-	12	34
Yuxi (YX)	-	-	14	-	21	4	39
Lincang (LC)	-	-	12	-	9	7	28
Wenshan (WS)	-	-	9	-	2	14	25
Chuxiong (CX)	6	-	4	-	6	10	26
Kunming (KM)	-	-	20	-	28	-	48
Total	27	67	114	4	83	57	352

**Table 2:** Primers and corresponding sequences used in this study.

Gene	Gene Name	Organism	Gene Bank acc. No.	Primer Sequence (5’–3’)	Product Size (bp)	Temperature
*Ef-1*	Elongation factor	*Pgt*	X73529.1	Ef137S: AAGCCGCATCCTTCGTTG Ef531A: TTGCCATCCGTCTTCTCG	395	51
*Mapk1*	Map kinase 1	*Pst*	HM535614.1	Map1351S: GTCGGTCGGGTGTATCCT Map1683A: GGTTCATCTTCGGGGTCA	332	53
*Cdc2*	Cyclin dependent kinase 2	*Pst*	GQ911579.1	Cdc28S: AAATCATCCACATCTGCTCCAC Cdc352A: TCCTACAAACCCCTCCAAAGGA	325	55
HSP	heat sock protein 90 kDa (hsp90)	*Pst*	AJIL01000023.1	Hsp2396S: TGCTCGTCACTGGTCAGTTC Hsp2680A: CGAAGAGGAGGACACTCAGG	285	52
UBA	ubiquitin-activating enzyme E1 (UBA)	*Pst*	AJIL01000094.1	Uba1715S: ACCCAAACCACGGAACCC Uba2088A: TCGCTCCAGCACCAACTA	374	59
UBC	ubiquitin-conjugating enzyme E2	*Pst*	AJIL01000007.1	Ubc279S: TTTGCGAATGGAGTATGG Ubc581A: GAGGGACTGACCTTTGAC	303	52

*Puccinia graminis* f. sp. *tritici* (*Pgt*), *Puccinia striiformis* f. sp. *tritici* (*Pst*), Elongation factor (EF-1), Map kinase 1 (MAPK), Cyclin-dependent kinase 2 (CDC2), heat shock protein 90 kDa (HSP), ubiquitin-activating enzyme E1 (UBA) and ubiquitin-conjugating enzyme E2 (UBC).

**Table 3:** SNP information of different locations in Yunnan.

Location	LJ	DH	BS	DL	QJ	ZT	YX	LC	WS	CX	KM	Total
SNP locus	26	34	18	27	24	26	26	23	33	26	25	42
Haplotype	25	24	19	30	23	30	31	22	22	22	37	220
Private. hap	13	18	10	20	16	17	21	12	13	15	26	181
Total	32	29	23	36	32	34	39	28	25	26	48	352

**Table 4:** Indices of molecular diversity in *Pst* population.

Regions	Haplotype Diversity (*Hd*)	Nucleotide Diversity (*Pi*)	Population Mutation Rate (*θ*)	Recombination Event (*Rm*)	Tajima’s *D*/*p*-Value	Fu’s *Fs*/*p*-Value
Lijiang (LJ)	0.98	3.91 × 10^−3^	4.42 × 10^−3^	6	−0.40263/0.4410	−16.332/0.000 **
Dehong (DH)	0.98	4.22 × 10^−3^	4.18 × 10^−3^	8	−0.15861/0.4530	−12.02127/0.000 **
Baoshan (BS)	0.980	3.92 × 10^−3^	3.34 × 10^−3^	7	0.63788/0.7890	−10.793/0.000 **
Dali (DL)	0.989	5.47 × 10^−3^	4.45 × 10^−3^	7	0.79275/0.8100	−19.035/0.000 **
Qujing (QJ)	0.946	5.95 × 10^−3^	4.08 × 10^−3^	8	1.61410/0.1095	−7.865/0.000 **
Zhaotong (ZT)	0.993	5.94 × 10^−3^	4.35 × 10^−3^	11	1.280431/0.9120	−19.836/0.000 **
Yuxi (YX)	0.972	5.05 × 10^−3^	4.21 × 10^−3^	9	0.680891/0.7940	−19.998/0.000 **
Lincang (LC)	0.968	5.49 × 10^−3^	4.04 × 10^−3^	7	1.278558/0.9210	−9.420/0.000 **
Wenshan (WS)	0.990	6.48 × 10^−3^	5.98 × 10^−3^	9	0.31318/0.6420	−10.373/0.000 **
Chuxiong (CX)	0.985	5.93 × 10^−3^	4.66 × 10^−3^	7	1.002346/0.8780	−10.233/0.000 **
Kunming (KM)	0.987	5.40 × 10^−3^	3.85 × 10^−3^	11	1.323307/0.9230	−24.891/0.000 **
Total	0.992	6.04 × 10^−3^	4.46 × 10^−3^	18	0.8238/0.77364	−369.901/0.000 **

** indicates statistically highly significant.

**Table 5:** *Gst* and *Nm* between Yuxi and other populations.

Parameter	Lijiang (LJ)	Dehong (DH)	Baoshan (BS)	Dali (DL)	Qujing (QJ)	Zhaotong (ZT)	Kunming (KM)	Lincang (LC)	Wenshan (WS)	Chuxiong (CX)	Among All Populations
*Gst*	0.0123	0.00875	0.01199	0.00819	0.02104	0.00634	0.00495	0.00307	0.0046	0.01126	0.01337
*Nm*	20.08	28.33	20.61	30.28	11.63	39.19	50.30	81.17	54.09	21.96	18.45

**Table 6:** AMOVA of *Pst* pathogens during the years from 2004 to 2015.

Source of Variation	df	Sum of Square	Variance Components	Percentage of Variation (%)	*p*-Value
Among groups	5	151.381	0.21516	4.71	0.11926
Among populations within groups	5	82.068	0.41509	9.09	0.00
Within populations	341	1342.014	3.93552	86.20	0.00
Total	351	1575.463	4.56578		

## Data Availability

Data used in the article are available in all Tables and Figures.

## Supplementary Materials

The following are available online at https://www.mdpi.com/article/10.3390/plants10081735/s1, Figure S1: The best number of groups among locations estimated by Evano test methods; Figure S2: The determination of the best number of clusters among 220 haplotypes by Evano test methods, Figure S3: Distinct groups among 11 locations; Table S1: Isolates collected from different counties of Yunnan and Sichuan Provinces; Table S2: Haplotypes and their SNP loci of *Pst* population; Table S3: Number of haplotypes and their distribution among the different locations.

## Abbreviations

AFLP	Amplified fragment length polymorphism
AMOVA	Analysis of molecular variance
CDC2	Cyclin-dependent kinase 2
CTAB	cetyltrimethylammonium bromide
EDTA	Ethylenediamine tetra-acetic acid
EF-1	Elongation factor
HSP	heat shock protein 90 kDa
MAPK	Map kinase 1
MCMC	Markov chain Monte Carlo (MCMC) methods comprise a class of algorithms for sampling from a probability distribution
MEGA	Molecular Evolutionary Genetics Analysis
PCA	Principal component analysis
PLS-DA	Partial Least-Squares Discriminant Analysis
*Pst*	*Puccinia striiformis* f. sp. *tritici*
RAPD	Random Amplification of Polymorphic DNA
SNPs	Single nucleotide polymorphisms
SSR	Simple Sequence Repeats
UBA	ubiquitin-activating enzyme E1
UBC	ubiquitin-conjugating enzyme E2
UPGMA	Unweighted Pair Group Method with Arithmetic Mean
