# Supplementary material for: Genetic Diversity of Wheat Stripe Rust Fungus Puccinia striiformis f. sp. tritici in Yunnan, China
Source: Plants (Basel). 2021 Aug 23;10(8):1735. doi: 10.3390/plants10081735 (PMC8399030; doi:10.3390/plants10081735)
Supplement: Supplementary file 1 [file plants-10-01735-s001.zip › plants-1305975-supplementary.pdf]

plants MDPI

# Supplementary Materials

**Table S1.** Isolates collected from different counties of Yunnan Province, China.

| Sl. No. | Isolate ID | Collection date | Location | Longitude | Latitude | Altitude (m) | Wheat cultivar |
|---|---|---|---|---|---|---|---|
| 1 | A8 | 2004.5.8 | Chuxiong | E101°34'10" | N25°02'16" | 1780 | Yunmai 42 |
| 2 | A9 | 2004.5.8 | Chuxiong | E101°34'10" | N25°02'16" | 1780 | Yunmai 42 |
| 3 | A10 | 2004.5.8 | Chuxiong | E101°34'10" | N25°02'16" | 1780 | Yunmai 42 |
| 4 | A12 | 2004.5.9 | Chuxiong | | | | Unknown |
| 5 | A16 | 2004.3.9 | Chuxiong | E101°34'10" | N25°02'16" | 1780 | Chu 22 to 4 |
| 6 | A17 | 2004.3.17 | Chuxiong | E101.58° | N25.32° | 1780 | Climbing 93-1 |
| 7 | A73 | 2004.5.25 | Lijiang | E100.25° | N26.86° | 2400 | Youth 55 |
| 8 | A74 | 2004.5.25 | Lijiang | E100.25° | N26.86° | 2400 | Youth 56 |
| 9 | A75 | 2004.5.25 | Lijiang | E100.25° | N26.86° | 2400 | Youth 57 |
| 10 | A76 | 2004.5.25 | Lijiang | E100.25° | N26.86° | 2400 | 79-16 |
| 11 | A77 | 2004.5.25 | Lijiang | E100.25° | N26.86° | 2400 | 79-16 |
| 12 | A78 | 2004.5.25 | Lijiang | E100.25° | N26.86° | 2400 | 79-16 |
| 13 | A79 | 2004.5.25 | Lijiang | E100.25° | N26.86° | 2400 | Yun Xuan 11-12 |
| 14 | A80 | 2004.5.25 | Lijiang | E100.25° | N26.86° | 2400 | Xiafan |
| 15 | A82 | 2004.5.14 | Lijiang | E100.25° | N26.86° | 2400 | 79-16 |
| 16 | A83 | 2004.5.14 | Lijiang | E100.25° | N26.86° | 2400 | 79-16 |
| 17 | A84 | 2004.5.14 | Lijiang | E100.25° | N26.86° | 2400 | Unknown |
| 18 | A85 | 2004.5.14 | Lijiang | E100.25° | N26.86° | 2400 | Unknown |
| 19 | A86 | 2004.5.14 | Lijiang | E100.25° | N26.86° | 2400 | Unknown |
| 20 | A88 | 2004.5.28 | Zhaotong | E104.86° | N27.42° | 1500 | Yunmai 19 |
| 21 | A90 | 2004.5.14 | Lijiang | E100.25° | N26.86° | 2400 | Limai 4 |
| 22 | A91 | 2004.5.14 | Lijiang | E100.25° | N26.86° | 2400 | Limai 5 |
| 23 | A92 | 2004.5.14 | Lijiang | E100.25° | N26.86° | 2400 | 79-16 |
| 24 | A97 | 2004.5.28 | Zhaotong | E104.86° | N27.42° | 1500 | Yunmai 19 |
| 25 | A98 | 2004.5.28 | Zhaotong | E104.86° | N27.42° | 1500 | Yunmai 20 |
| 26 | A104 | 2004.5.28 | Zhaotong | E104.86° | N27.42° | 1500 | Abo |
| 27 | A111 | 2004.5.29 | Zhaotong | E104.86° | N27.42° | 1500 | Abo |
| 28 | B3 | 2008.2.26 | Dehong | E98°18′ | N24°45′ | 1020 | Bald wheat |
| 29 | B5 | 2008.2.26 | Dehong | E98°18′ | N24°45′ | 1020 | Mianyang 19 |
| 30 | B6 | 2008.2.26 | Dehong | E97.8° | N 24.9° | | Mianyang 11 |
| 31 | B8 | 2008.4.19 | Baoshan | E98°55′ | N24°35′ | | Mianyang 11 |
| 32 | B10 | 2008.4.19 | Baoshan | E98°55′ | N24°35′ | | Unknown |
| 33 | B11 | 2008.4.19 | Baoshan | E98°55′ | N24°35′ | | Unknown |
| 34 | B13 | 2008.2.29 | Baoshan | E99°8′47″ | N25°2′47″ | 1670 | Accession 3 |
| 35 | B14 | 2008.2.29 | Baoshan | E99°8′47″ | N25°2′47″ | 1670 | Yunza 5 |
| 36 | B15 | 2008.2.29 | Baoshan | E99°8′47″ | N25°2′47″ | 1670 | 93-12 |
| 37 | B16 | 2008.2.29 | Baoshan | E99°8′47″ | N25°2′47″ | 1670 | Unknown |
| 38 | B17 | 2008.4.9 | Baoshan | E99°8′47″ | N25°2′47″ | 1670 | Unknown |
| 39 | B18 | 2008.4.9 | Baoshan | E99°8′47″ | N25°2′47″ | 1670 | Accession 3 |
| 40 | B19 | 2008.4.9 | Baoshan | E99°8′47″ | N25°2′47″ | 1670 | Chumai 10 |
| 41 | B20 | 2008.5.2 | Baoshan | E99°8′47″ | N25°2′47″ | 1670 | Yunmai 52 |
| 42 | B21 | 2008.5.2 | Baoshan | E99°8′47″ | N25°2′47″ | 1670 | Yunza 5 |

| Sl. No. | Isolate ID | Collection date | Location | Longitude | Latitude | Alti-tude (m) | Wheat cultivar |
|---|---|---|---|---|---|---|---|
| 43 | B22 | 2008.5.2 | Baoshan | E99°8′47″ | N25°2′47″ | 1670 | Yimai 1 |
| 44 | B23 | 2008.5.2 | Baoshan | E99°8′47″ | N25°2′47″ | 1670 | Yunmai 52 |
| 45 | B24 | 2008.5.2 | Baoshan | E99°8′47″ | N25°2′47″ | 1670 | 1928848 |
| 46 | B26 | 2008.5.2 | Baoshan | E99°8′47″ | N25°2′47″ | 1670 | German tons of wheat |
| 47 | B27 | 2008.5.2 | Dali | E100°10′ | N 25°40′ | 1990 | Fengmai 13 |
| 48 | B28 | 2008.5.2 | Dali | E100°10′ | N 25°40′ | 1990 | Fengmai 24 |
| 49 | B29 | 2008.5.2 | Dali | E100°10′ | N 25°40′ | 1990 | Feng Mai 31-27-18-7-3 |
| 50 | B30 | 2008.5.2 | Dali | E100°10′ | N 25°40′ | 1990 | Feng Mai 33 |
| 51 | B31 | 2008.5.2 | Dali | E100°10′ | N 25°40′ | 1990 | Fengmai 35 |
| 52 | B32 | 2008.5.2 | Dali | E100°10′ | N 25°40′ | 1990 | Li 03-6 |
| 53 | B33 | 2008.5.2 | Dali | E99°51′ | N 25°20′ | 1990 | Yimai 1 |
| 54 | B35 | 2008.3.19 | Dali | E100°10′ | N 25°40′ | 1990 | Feng Mai 35 |
| 55 | B37 | 2008.3.19 | Dali | E100°10′ | N 25°40′ | 1990 | Feng Mai 33 |
| 56 | B38 | 2008.3.20 | Dali | E99°51′ | N 25°20′ | 1990 | Yimai 1 |
| 57 | B39 | 2008.2.17 | Dali | E100°26′ | N 24°51′ | 1380 | Xindu 762 |
| 58 | B41 | 2008.2.17 | Dali | E100°26′ | N 24°51′ | 1650 | Unknown |
| 59 | B42 | 2008.2.17 | Dali | E100°26′ | N 24°51′ | 1650 | Unknown |
| 60 | B44 | 2008.4.17 | Qujing | E103°45′ | N26°13′ | 2000 | Jing 06-4 |
| 61 | B45 | 2008.4.17 | Qujing | E103°45′ | N26°13′ | 2000 | Sichuan wheat 107 |
| 62 | B46 | 2008.4.17 | Qujing | E103°45′ | N26°13′ | 2000 | Yunmai 42 |
| 63 | B47 | 2008.4.17 | Qujing | E103°45′ | N26°13′ | 2000 | Yi 2003-27 |
| 64 | B48 | 2008.4.17 | Qujing | E103°45′ | N26°13′ | 2000 | Yi Mai No. 10 |
| 65 | B49 | 2008.4.17 | Qujing | E103°45′ | N26°13′ | 2000 | Wenmai 11. |
| 66 | B51 | 2008.4.17 | Qujing | E103°45′ | N26°13′ | 2000 | Chu 0234 |
| 67 | B52 | 2008.4.17 | Qujing | E103°45′ | N26°13′ | 2000 | Pro 04j59 |
| 68 | B54 | 2008.4.17 | Qujing | E103°45′ | N26°13′ | 2000 | Chuannong 16 |
| 69 | B55 | 2008.4.17 | Qujing | E103°45′ | N26°13′ | 2000 | Jingmai 12 |
| 70 | B56 | 2008.4.17 | Qujing | E103°45′ | N26°13′ | 2000 | 06D6-6 |
| 71 | B57 | 2008.4.17 | Qujing | E103°45′ | N26°13′ | 2000 | Chuannong 107 |
| 72 | B58 | 2008.4.17 | Qujing | E103°45′ | N26°13′ | 2000 | Fengmai 24 |
| 73 | B59 | 2008.4.17 | Qujing | E103°45′ | N26°13′ | 2000 | Chuannong 16 |
| 74 | B61 | 2008.4.17 | Qujing | E103°45′ | N26°13′ | 2000 | Yi 2003-27 |
| 75 | B62 | 2008.4.17 | Qujing | E103°45′ | N26°13′ | 2000 | Jing 06-4 |
| 76 | B63 | 2008.4.17 | Qujing | E103°45′ | N26°13′ | 2000 | Chu 0234 |
| 77 | B64 | 2008.4.17 | Qujing | E103°45′ | N26°13′ | 2000 | Jingmai 12 |
| 78 | B65 | 2008.4.17 | Qujing | E103°45′ | N26°13′ | 2000 | Yimai 10 |
| 79 | B66 | 2008.4.17 | Qujing | E103°45′ | N26°13′ | 2000 | 02D2-282 |
| 80 | B68 | 2008.4.17 | Qujing | E103°30′ | N26.7° | 1820 | Local wheat (Mianyang series) |
| 81 | B69 | 2008.4.17 | Qujing | E103°54′ | N26°5′ | 2060 | Local wheat (Mianyang series) |
| 82 | B97 | 2008.5.14 | Zhaotong | E104°25′ | N27°25′ | 1500 | Piedmont wheat |
| 83 | B98 | 2008.5.14 | Zhaotong | E104°25 | ′N27°25′ | 1500 | Dam area wheat |
| 84 | B99 | 2008.5.14 | Zhaotong | E104°25′ | N27°25′ | 1500 | Beijing wheat |
| 85 | B100 | 2008.5.14 | Zhaotong | E104°25′ | N27°25′ | 1500 | Chuanmai 107 |
| 86 | B102 | 2008.5.14 | Zhaotong | E104°25′ | N27°25′ | 1500 | McNair 701 |
| 87 | B103 | 2008.5.14 | Zhaotong | E104°25′ | N27°25′ | 1500 | Stone 4185 |
| 88 | B104 | 2008.5.14 | Zhaotong | E104°25′ | N27°25′ | 1500 | Jinmai 47 |
| 89 | B105 | 2008.5.14 | Zhaotong | E104°25′ | N27°25′ | 1500 | Zheng Mai 9023 |
| 90 | B106 | 2008.5.14 | Zhaotong | E104°25′ | N27°25′ | 1500 | Yangmai 158 |
| 91 | B107 | 2008.5.14 | Zhaotong | E104°25′ | N27°25′ | 1500 | West Peak 20 |
| 92 | B108 | 2008.5.14 | Zhaotong | E104°25′ | N27°25′ | 1500 | Khapli/8cc |

| Sl. No. | Isolate ID | Collection date | Location | Longitude | Latitude | Alti-tude (m) | Wheat cultivar |
|---|---|---|---|---|---|---|---|
| 93 | B109 | 2008.5.14 | Zhaotong | E104°25′ | N27°25′ | 1500 | Unknown |
| 94 | B110 | 2008.5.14 | Zhaotong | E104°25′ | N27°25′ | 1500 | Jinmai 54 |
| 95 | C1 | 2011.4.6 | Yuxi | E102.52° | N24.35° | 1630 | Piedmont wheat |
| 96 | C2 | 2011.4.6 | Yuxi | E102.52° | N24.35° | 1630 | Aurora |
| 97 | C3 | 2011.4.6 | Yuxi | E102.52° | N24.35° | 1630 | F49-71 |
| 98 | C4 | 2011.4.6 | Yuxi | E102.52° | N24.35° | 1630 | Lumai 23 |
| 99 | C5 | 2011.4.6 | Yuxi | E102.52° | N24.35° | 1630 | Mingxian 169*6/Yr10 |
| 100 | C6 | 2011.4.7 | Yuxi | E102.52° | N24.35° | 1630 | Mingxian 169 |
| 101 | C7 | 2011.4.7 | Yuxi | E102.52° | N24.35° | 1630 | chancellor |
| 102 | C8 | 2011.4.7 | Yuxi | E102.52° | N24.35° | 1630 | Yangmai 158 |
| 103 | C9 | 2011.4.7 | Yuxi | E102.52° | N24.35° | 1630 | Avocet S*6/Yr7 |
| 104 | C10 | 2011.4.7 | Yuxi | E102.52° | N24.35° | 1630 | Avocet S*6/Yr26 |
| 105 | C11 | 2011.4.7 | Yuxi | E102.52° | N24.35° | 1630 | Avocet R YrA |
| 106 | C12 | 2011.4.7 | Yuxi | E102.52° | N24.35° | 1630 | BtSr30 Wst |
| 107 | C13 | 2011.4.7 | Yuxi | E102.52° | N24.35° | 1630 | Compare |
| 108 | C14 | 2011.4.7 | Yuxi | E102.52° | N24.35° | 1630 | Stone 4185 |
| 109 | C27 | 2011.2.20 | Dehong | E98°33.653 | N24°17.454 | 1230 | Yunmai 38 |
| 110 | C28 | 2011.3.8 | Dehong | E98°35'07" | N24°28'25" | 906 | Nanda 2419 |
| 111 | C30 | 2011.3.8 | Dehong | E98°35'07" | N24°28'25" | 906 | Yumai 49 |
| 112 | C31 | 2011.3.8 | Dehong | E98°35'07" | N24°28'25" | 906 | Jinmai 47 |
| 113 | C32 | 2011.3.8 | Dehong | E98°35'07" | N24°28'25" | 906 | Jin Tai 170 |
| 114 | C33 | 2011.3.8 | Dehong | E98°35'07" | N24°28'25" | 906 | Mingxian 169 |
| 115 | C34 | 2011.3.8 | Dehong | E98°35'07" | N24°28'25" | 906 | Morocco |
| 116 | C35 | 2011.3.8 | Dehong | E98°35'07" | N24°28'25" | 906 | Line E |
| 117 | C36 | 2011.3.8 | Dehong | E98°35'07" | N24°28'25" | 906 | W2691 SrTt-1 |
| 118 | C37 | 2011.3.8 | Dehong | E98°35'07" | N24°28'25" | 906 | Avocet S |
| 119 | C38 | 2011.3.8 | Dehong | E98°35'07" | N24°28'25" | 906 | Mianyang 11 |
| 120 | C39 | 2011.3.8 | Dehong | E98°35'07" | N24°28'25" | 906 | Avocet S*6/Yr1 |
| 121 | C40 | 2011.3.8 | Dehong | E98°35'07" | N24°28'25" | 906 | Avocet S*6/Yr6 |
| 122 | C41 | 2011.3.8 | Dehong | E98°35'07" | N24°28'25" | 906 | Avocet S*6/Yr7 |
| 123 | C42 | 2011.3.8 | Dehong | E98°35'07" | N24°28'25" | 906 | Verstein Sr9e |
| 124 | C43 | 2011.3.8 | Dehong | E98°35'07" | N24°28'25" | 906 | Beijing Double 16 |
| 125 | C44 | 2011.3.8 | Dehong | E98°35'07" | N24°28'25" | 906 | Orou |
| 126 | C45 | 2011.3.8 | Dehong | E98°35'07" | N24°28'25" | 906 | Chuan Yu 55871 |
| 127 | C46 | 2011.3.8 | Dehong | E98°35'07" | N24°28'25" | 906 | Zheng Mai 9023 |
| 128 | C47 | 2011.3.4 | Lincang | E100°05'55" | N24°19'47" | 1660 | Linmai 6 |
| 129 | C48 | 2011.3.4 | Lincang | E100°05'55" | N24°19'47" | 1660 | Linmai 6 |
| 130 | C49 | 2011.3.4 | Lincang | E100°06'15" | N24°21'01" | 1520 | Linmai 15 |
| 131 | C50 | 2011.3.4 | Lincang | E100°01'46" | N24°31'38" | 1406 | Linmai 6 |
| 132 | C51 | 2011.3.4 | Lincang | E99°58'33" | N24°33'02" | 1500 | Linmai 6 |
| 133 | C52 | 2011.3.4 | Lincang | E99°58'00" | N24°33'30" | 1540 | Linmai 6 |
| 134 | C53 | 2011.3.4 | Lincang | E100°01'46" | N24°31'38" | 1406 | Linmai 6 |
| 135 | C54 | 2011.3.4 | Lincang | E100°18'41" | N24°00'27" | 1700 | Linmai 6 |
| 136 | C55 | 2011.3.4 | Lincang | E100°18'41" | N24°00'27" | 1700 | Choose 188 |
| 137 | C56 | 2011.3.4 | Lincang | E100°16'23" | N24°06'40" | 1435 | Choose 188 |
| 138 | C57 | 2011.3.3 | Lincang | E100°14'07" | N24°22'54" | 1260 | Linmai 6 |
| 139 | C58 | 2011.3.3 | Lincang | E100°14'14" | N23°53'31" | 1460 | Linmai 10 |
| 140 | C59 | 2011.3.24 | Dali | E100°18'22" | N25°35'23" | 1990 | Linmai 6 |
| 141 | C60 | 2011.3.25 | Dali | E100°28'06" | N25°20'22" | 1670 | Fengmai 35 |
| 142 | C61 | 2011.3.25 | Dali | E100°28'06" | N25°20'22" | 1670 | Mingxian 169 |

| Sl. No. | Isolate ID | Collection date | Location | Longitude | Latitude | Alti-tude (m) | Wheat cultivar |
|---|---|---|---|---|---|---|---|
| 143 | C62 | 2011.3.25 | Dali | E100°28'06" | N25°20'22" | 1670 | Avocet S*6/Yr27 |
| 144 | C63 | 2011.3.25 | Dali | E100°28'06" | N25°20'22" | 1670 | Carsten v |
| 145 | C64 | 2011.3.25 | Dali | E100°28'06" | N25°20'22" | 1670 | Mingxian 169*6/Yr10 |
| 146 | C65 | 2011.3.25 | Dali | E100°28'06" | N25°20'22" | 1670 | Qian Baode |
| 147 | C66 | 2011.3.25 | Dali | E100°28'06" | N25°20'22" | 1670 | Baofeng 104 |
| 148 | C67 | 2011.3.25 | Dali | E100°28'06" | N25°20'22" | 1670 | Jimai 38 |
| 149 | C68 | 2011.3.25 | Dali | E100°28'06" | N25°20'22" | 1670 | Jinmai 47 |
| 150 | C69 | 2011.3.25 | Dali | E100°28'06" | N25°20'22" | 1670 | Zheng Mai 9023 |
| 151 | C71 | 2011.3.25 | Dali | E100°28'06" | N25°20'22" | 1670 | Chinese 166 |
| 152 | C72 | 2011.3.25 | Dali | E100°28'06" | N25°20'22" | 1670 | McNair 701 |
| 153 | C73 | 2011.3.23 | Baoshan | E99°11'23" | N25°08'55" | 1660 | Mingxian 169 |
| 154 | C74 | 2011.3.23 | Baoshan | E99°11'23" | N25°08'55" | 1660 | S39 |
| 155 | C75 | 2011.3.23 | Baoshan | E99°11'23" | N25°08'55" | 1660 | Regional Test Variety 2 |
| 156 | C76 | 2011.3.23 | Baoshan | E99°11'23" | N25°08'55" | 1660 | Regional test variety 3 |
| 157 | C77 | 2011.3.23 | Baoshan | E99°11'23" | N25°08'55" | 1660 | Regional Test Variety 5 |
| 158 | C78 | 2011.3.23 | Baoshan | E99°11'23" | N25°08'55" | 1660 | Regional test variety 9 |
| 159 | C79 | 2011.3.23 | Baoshan | E99°09'05" | N25°02'28" | 1670 | Bald wheat |
| 160 | C80 | 2011.4.8 | Lijiang | E100°16'59" | N26°36'13" | 2480 | New 16 |
| 161 | C81 | 2011.4.8 | Lijiang | E100°16'59" | N26°36'13" | 2480 | New 16 |
| 162 | C82 | 2011.4.8 | Lijiang | E100°16'59" | N26°36'13" | 2480 | New 16 |
| 163 | C83 | 2011.4.8 | Lijiang | E100°16'59" | N26°36'13" | 2480 | New 16 |
| 164 | C84 | 2011.4.8 | Lijiang | E100°16'59" | N26°36'13" | 2480 | New 16 |
| 165 | C85 | 2011.4.8 | Lijiang | E99°23'-101°31' | N25°91'-27°56' | 1980 | 79-16 |
| 166 | C86 | 2011.4.8 | Lijiang | E99°23'-101°31' | N25°91'-27°56' | 1980 | 79-16 |
| 167 | C87 | 2011.4.8 | Lijiang | E100°16'59" | N26°36'13" | 2480 | New 16 |
| 168 | C88 | 2011.4.8 | Lijiang | E100°16'59" | N26°36'13" | 2480 | New 16 |
| 169 | C89 | 2011.4.8 | Lijiang | E100°16'59" | N26°36'13" | 2480 | New 16 |
| 170 | C91 | 2011.4.8 | Lijiang | E100°16'59" | N26°36'13" | 2480 | New 16 |
| 171 | C92 | 2011.4.8 | Lijiang | E100°16'59" | N26°36'13" | 2480 | New 16 |
| 172 | C93 | 2011.3.28 | Wenshan | E105.09° | N24.05 ° | 1280 | Unknown |
| 173 | C94 | 2011.3.24 | Wenshan | E104.71° | N 23.12° | 1410 | Unknown |
| 174 | C95 | 2011.3.28 | Wenshan | E104.19° | N24.03° | 1460 | Unknown |
| 175 | C96 | 2011.3.28 | Wenshan | E104.19° | N24.03° | 1460 | Unknown |
| 176 | C98 | 2011.3.28 | Wenshan | E104.19° | N24.03° | 1450 | Unknown |
| 177 | C99 | 2011.4.15 | Wenshan | E104.35° | N23.62° | 1480 | Bald wheat |
| 178 | C100 | 2011.4.15 | Wenshan | E104.35° | N23.62° | 1480 | Bald wheat |
| 179 | C101 | 2011.4.15 | Wenshan | E104.35° | N23.62° | 1480 | Cloud Wheat Series |
| 180 | C102 | 2011.4.19 | Wenshan | E104.35° | N23.62° | 1540 | Unknown |
| 181 | C103 | 2011.4.11 | Chuxiong | E101°34'10" | N25°02'16" | 1780 | 802-2 |
| 182 | C104 | 2011.4.11 | Chuxiong | E101°34'10" | N25°02'16" | 1780 | Mingxian 169 |
| 183 | C105 | 2011.4.11 | Chuxiong | E101°34'10" | N25°02'16" | 1780 | Mingxian 169 |
| 184 | C106 | 2011.4.11 | Chuxiong | E101°34'10" | N25°02'16" | 1780 | Yunmai 42 |
| 185 | C107 | 2011.4.7 | Kunming | E 102°51'13" | N 25°06'43" | 2145 | Mingxian 169 |
| 186 | C108 | 2011.4.7 | Kunming | E 102°51'13" | N 25°06'43" | 2145 | Mingxian 169 |
| 187 | C109 | 2011.4.7 | Kunming | E 102°51'13" | N 25°06'43" | 2145 | Mingxian 169*6/Yr10 |
| 188 | C110 | 2011.4.7 | Kunming | E 102°51'13" | N 25°06'43" | 2145 | Morocco |
| 189 | C111 | 2011.4.7 | Kunming | E 102°51'13" | N 25°06'43" | 2145 | Chancellor |
| 190 | C112 | 2011.4.7 | Kunming | E 102°51'13" | N 25°06'43" | 2145 | Line E |
| 191 | C113 | 2011.4.7 | Kunming | E 102°51'13" | N 25°06'43" | 2145 | Ji Mai 38 |
| 192 | C114 | 2011.4.7 | Kunming | E 102°51'13" | N 25°06'43" | 2145 | Jinmai 54 |

| Sl. No. | Isolate ID | Collection date | Location | Longitude | Latitude | Altitude (m) | Wheat cultivar |
|---|---|---|---|---|---|---|---|
| 193 | C115 | 2011.4.7 | Kunming | E 102°51'13" | N 25°06'43" | 2145 | Jin Tai 170 |
| 194 | C116 | 2011.4.7 | Kunming | E 102°51'13" | N 25°06'43" | 2145 | Avocet S*6/Yr7 |
| 195 | C117 | 2011.4.7 | Kunming | E 102°51'13" | N 25°06'43" | 2145 | Jinmai 47 |
| 196 | C118 | 2011.4.7 | Kunming | E 102°51'13" | N 25°06'43" | 2145 | Yumai 18 |
| 197 | C119 | 2011.4.7 | Kunming | E 102°51'13" | N 25°06'43" | 2145 | McMair 701 |
| 198 | C120 | 2011.4.7 | Kunming | E 102°51'13" | N 25°06'43" | 2145 | Aurora |
| 199 | C121 | 2011.4.7 | Kunming | E 102°51'13" | N 25°06'43" | 2145 | Yumai 49 |
| 200 | C123 | 2011.4.7 | Kunming | E 102°51'13" | N 25°06'43" | 2145 | Avocet S*6/Yr9 |
| 201 | C125 | 2011.2.21 | Kunming | E 102°51'13" | N 25°06'43" | 2145 | Mingxian 169 |
| 202 | C127 | 2011.4.7 | Kunming | E 102°51'13" | N 25°06'43" | 2145 | Stone 4185 |
| 203 | C128 | 2011.4.7 | Kunming | E 102°51'13" | N 25°06'43" | 2145 | Jingdong 8 |
| 204 | C129 | 2011.4.7 | Kunming | E 102°51'13" | N 25°06'43" | 2145 | Unknown |
| 205 | C130 | 2011.4.20 | Zhaotong | E103.54° | N27.21° | 1550 | Zhaomai 1 |
| 206 | C131 | 2011.4.20 | Zhaotong | E103.54° | N27.21° | 1450 | Zhaomai 1 |
| 207 | C132 | 2011.4.20 | Zhaotong | E103.54° | N27.21° | 1450 | Yunmai 42 |
| 208 | C133 | 2011.4.18 | Zhaotong | E103.91° | N27.74° | 950 | Hanging 19 |
| 209 | D19 | 2012.4.5 | Lijiang | E100°03'45" | N26°44'51" | 2780 | Mingxian 169 |
| 210 | D20 | 2012.4.5 | Lijiang | E100°06'53" | N26°49'31" | 2468 | Mingxian 169 |
| 211 | D21 | 2012.4.5 | Lijiang | E100°11'43" | N26°52'53" | 2400 | 79-16 |
| 212 | D22 | 2012.4.5 | Lijiang | E100°11'43" | N26°52'53" | 2400 | 79-16 |
| 213 | M1 | 2014.3.25 | Qujing | | | 1920 | Unknown |
| 214 | M2 | 2014.3.25 | Qujing | | | 1920 | Unknown |
| 215 | M3 | 2014.3.26 | Qujing | 100°6'12" | 23°57'1" | 1460 | Dianmai 23 |
| 216 | M4 | 2014.3.26 | Qujing | 100°6'12" | 23°57'1" | 1460 | Clouds 11 |
| 217 | M5 | 2014.3.16 | Qujing | 103°51'14" | 25°22'24" | 1865 | Chuanmai 107 |
| 218 | M6 | 2014.3.16 | Qujing | 103°43.07' | 27°19.49' | 1865 | Chuanmai 107 |
| 219 | M7 | 2014.3.16 | Qujing | 103°43.07' | 27°19.49' | 1860 | Yimai 1 |
| 220 | M8 | 2014.3.26 | Lincang | 100°6'12" | 23°57'1" | 1460 | Yunmai 53 |
| 221 | M9 | 2014.3.26 | Lincang | 100°6'12" | 23°57'1" | 1460 | Unknown |
| 222 | M10 | 2014.3.26 | Lincang | 100°6'12" | 23°57'1" | 1460 | DE 10132 |
| 223 | M11 | 2014.3.26 | Lincang | 100°6'12" | 23°57'1" | 1460 | Yunmai 10 |
| 224 | M12 | 2014.3 | Wenshan | | | 1400 | Wenmai 8 |
| 225 | M13 | 2014.3.26 | Lincang | 100°6'12" | 23°57'1" | 1460 | Cloud 124-4 |
| 226 | M14 | 2014.3.26 | Lincang | 100°6'12" | 23°57'1" | 1460 | Linmai 6 |
| 227 | M15 | 2014.3 | Wenshan | | | 1400 | Wenmai 8 |
| 228 | M16 | 2014.3.26 | Lincang | 100°6'12" | 23°57'1" | 1460 | Linmai 20 |
| 229 | M17 | 2014.4.1 | Yuxi | 102°29'06" | 24°19'27" | 1631 | F49-71 |
| 230 | M19 | 2014.4.1 | Yuxi | 102°29'06" | 24°19'27" | 1631 | WF14TN-261 |
| 231 | M20 | 2014.4.1 | Yuxi | 102°29'06" | 24°19'27" | 1631 | Longyuan 935 |
| 232 | M21 | 2014.4.1 | Yuxi | 102°29'06" | 24°19'27" | 1631 | Guinong 22 |
| 233 | M23 | 2014.4.1 | Yuxi | 102°29'06" | 24°19'27" | 1631 | Unknown |
| 234 | M24 | 2014.4.1 | Yuxi | 102°29'06" | 24°19'27" | 1631 | Unknown |
| 235 | M26 | 2014.4.1 | Yuxi | 102°29'06" | 24°19'27" | 1631 | Jinmai 54 |
| 236 | M27 | 2014.4.1 | Yuxi | 102°29'06" | 24°19'27" | 1631 | Chancellor |
| 237 | M28 | 2014.4.1 | Yuxi | 102°29'06" | 24°19'27" | 1631 | Compair |
| 238 | M29 | 2014.4.1 | Yuxi | 102°29'06" | 24°19'27" | 1631 | Nanda 2419 |
| 239 | M30 | 2014.4.1 | Yuxi | 102°29'06" | 24°19'27" | 1631 | Unknown |
| 240 | M31 | 2014.4.1 | Yuxi | 102°29'06" | 24°19'27" | 1631 | Jin Tai 170 |
| 241 | M32 | 2014.4.1 | Yuxi | 102°29'06" | 24°19'27" | 1631 | Mingxian 169 |
| 242 | M33 | 2014.4.1 | Yuxi | 102°29'06" | 24°19'27" | 1631 | Unknown |

| Sl. No. | Isolate ID | Collection date | Location | Longitude | Latitude | Alti-tude (m) | Wheat cultivar |
|---|---|---|---|---|---|---|---|
| 243 | M34 | 2014.3.12 | Chuxiong | | | | Chuanmai 107 |
| 244 | M35 | 2014.3.13 | Chuxiong | | | | Morrocco |
| 245 | M36 | 2014.3.13 | Chuxiong | | | | Unknown |
| 246 | M37 | 2014.3.12 | Chuxiong | | | | Unknown |
| 247 | M38 | 2014.3.12 | Chuxiong | | | | Unknown |
| 248 | M39 | 2014.3.13 | Chuxiong | | | | Unknown |
| 249 | M40 | 2014.3.26 | Dehong | 98°30'37" | 24°17'17" | 1260 | Line E/Kavkaz |
| 250 | M41 | 2014.3.26 | Dehong | 98°30'37" | 24°17'17" | 1260 | Line E/Kavkaz |
| 251 | M42 | 2014.3.18 | Dali | | | 1960 | Bald wheat |
| 252 | M43 | 2014.3.21 | Dali | | | 1920 | Unknown |
| 253 | M44 | 2014.3.21 | Dali | | | 1920 | Unknown |
| 254 | M45 | 2014.4.1 | Yuxi | 102°29'06" | 24°19'27" | 1631 | Compair |
| 255 | M46 | 2014.3.18 | Dali | | | 1960 | Bald wheat |
| 256 | M47 | 2014.3.18 | Dali | | | 1960 | Bald wheat |
| 257 | M49 | 2014.4.1 | Yuxi | 102°29'06" | 24°19'27" | 1631 | Nanda 2419 |
| 258 | M50 | 2014.4.1 | Yuxi | 102°29'06" | 24°19'27" | 1631 | Zheng Mai 9023 |
| 259 | M52 | 2014.4.1 | Yuxi | 102°29'06" | 24°19'27" | 1631 | Stone 4185 |
| 260 | M53 | 2014.4.1 | Yuxi | 102°29'06" | 24°19'27" | 1631 | Ji Mai 38 |
| 261 | M54 | 2014.4.2 | Kunming | 103°02'10" | 25°18'29" | 1905 | Induce line |
| 262 | M55 | 2014.4.1 | Yuxi | 102°29'06" | 24°19'27" | 1631 | Heines Peko |
| 263 | M57 | 2014.4.1 | Yuxi | 102°29'06" | 24°19'27" | 1631 | Jinmai 47 |
| 264 | M58 | 2014.3.21 | Dali | | | 1920 | Unknown |
| 265 | M59 | 2014.4.2 | Kunming | 103°02'10" | 25°18'29" | 1905 | Induce line |
| 266 | M60 | 2014.4.2 | Kunming | 103°02'10" | 25°18'29" | 1905 | Induce line |
| 267 | M62 | 2014.3.18 | Dali | | | 1960 | Bald wheat |
| 268 | M63 | 2014.3.21 | Dali | | | 1920 | Unknown |
| 269 | M64 | 2014.3.18 | Dali | | | 1960 | Bald wheat |
| 270 | M65 | 2014.3.21 | Dali | | | 1920 | Unknown |
| 271 | M67 | 2014.4.2 | Kunming | 103°02'10" | 25°18'29" | 1905 | Induce line |
| 272 | M68 | 2014.4.9 | Kunming | 102°51'12" | 25°07'11" | 2140 | Avocet S*6/Yr7 |
| 273 | M69 | 2014.3.5 | Kunming | | | | Low-generation materials |
| 274 | M70 | 2014.4.2 | Kunming | 103°02'10" | 25°18'29" | 1905 | Induce line |
| 275 | M72 | 2014.3.5 | Kunming | | | | Unknown |
| 276 | M73 | 2014.4.2 | Kunming | 103°02'10" | 25°18'29" | 1905 | Induce line |
| 277 | M74 | 2014.4.2 | Kunming | 103°02'10" | 25°18'29" | 1905 | Induce line |
| 278 | M76 | 2014.4.2 | Kunming | 103°02'10" | 25°18'29" | 1905 | Induce line |
| 279 | M77 | 2014.4.2 | Kunming | 103°02'10" | 25°18'29" | 1905 | Induce line |
| 280 | M78 | 2014.4.2 | Kunming | 103°02'10" | 25°18'29" | 1905 | Induce line |
| 281 | M79 | 2014.4.2 | Kunming | 103°02'10" | 25°18'29" | 1905 | Induce line |
| 282 | M80 | 2014.4.2 | Kunming | 103°02'10" | 25°18'29" | 1905 | nduce line |
| 283 | M81 | 2014.3.5 | Kunming | | | | Unknown |
| 284 | M82 | 2014.3.5 | Kunming | | | | CA0389 |
| 285 | M83 | 2014.4.2 | Kunming | 103°02'10" | 25°18'29" | 1905 | Induce line |
| 286 | M84 | 2014.4.2 | Kunming | 103°02'10" | 25°18'29" | 1905 | Induce line |
| 287 | M85 | 2014.3.5 | Kunming | | | | Unknown |
| 288 | M86 | 2014.4.2 | Kunming | 103°02'10" | 25°18'29" | 1905 | Induce line |
| 289 | M87 | 2014.3.5 | Kunming | | | | Unknown |
| 290 | M88 | 2014.3.5 | Kunming | | | | CA0389 |
| 291 | M89 | 2014.3.5 | Kunming | | | | D2-71 |
| 292 | M90 | 2014.4.2 | Kunming | 103°02'10" | 25°18'29" | 1905 | Induce line |

| Sl. No. | Isolate ID | Collection date | Location | Longitude | Latitude | Alti-tude (m) | Wheat cultivar |
|---|---|---|---|---|---|---|---|
| 293 | M91 | 2014.4.2 | Kunming | 103°02'10" | 25°18'29" | 1905 | Unknown |
| 294 | M93 | 2014.4.2 | Kunming | 103°02'10" | 25°18'29" | 1905 | Dias 2 |
| 295 | M94 | 2014.4.2 | Kunming | 103°02'10" | 25°18'29" | 1905 | Induce line |
| 296 | N1 | 2015.3.19 | Zhaotong | | | | Unknown |
| 297 | N2 | 2015.3.19 | Zhaotong | | | | Local wheat |
| 298 | N3 | 2015.3.19 | Zhaotong | | | | Yangmai 158 |
| 299 | N4 | 2015.3.19 | Zhaotong | | | | Zheng Mai 9023 |
| 300 | N5 | 2015.3.19 | Zhaotong | | | | Triumph 64 |
| 301 | N6 | 2015.3.19 | Zhaotong | | | | Mingxian 169*6/ Yr10 |
| 302 | N7 | 2015.3.9 | Wenshan | E103°34' | N23°45' | 1450 | Chuanmai 107 |
| 303 | N8 | 2015.3.9 | Wenshan | E103°34' | N23°45' | 1450 | Chuanmai 107 |
| 304 | N9 | 2015.3.9 | Wenshan | | | 1720 | Old variety bald wheat |
| 305 | N10 | 2015.3.9 | Wenshan | E103°34' | N23°45' | 1450 | Chuanmai 107 |
| 306 | N11 | 2015.3.9 | Wenshan | E103°34' | N24°28' | 1470 | Shimai 001 |
| 307 | N12 | 2015.3.9 | Wenshan | E103°34' | N23°45' | 1450 | Chuanmai 107 |
| 308 | N13 | 2015.3.9 | Wenshan | E103°34' | N23°45' | 1450 | Chuanmai 107 |
| 309 | N14 | 2015.3.9 | Wenshan | E103°34' | N23°45' | 1450 | Chuanmai 107 |
| 310 | N15 | 2015.3.9 | Wenshan | E103°34' | N23°45' | 1450 | Chuanmai 107 |
| 311 | N16 | 2015.3.9 | Wenshan | E103°34' | N24°28' | 1470 | Shimai 001 |
| 312 | N17 | 2015.3.9 | Wenshan | E103°34' | N24°28' | 1470 | Shimai 001 |
| 313 | N18 | 2015.3.9 | Wenshan | E103°34' | N24°28' | 1470 | Shimai 001 |
| 314 | N19 | 2015.3.9 | Wenshan | E103°34' | N24°28' | 1470 | Shimai 001 |
| 315 | N20 | 2015.3.5 | Yuxi | E102°13'19" | N24°40'7" | 1500 | Yunmai 42 |
| 316 | N21 | 2015.3.9 | Wenshan | E103°34' | N23°45' | 1450 | Chuanmai 107 |
| 317 | N22 | 2015.3.12 | Dehong | E98°58' | N24°43' | 914 | C99 |
| 318 | N23 | 2015.3.12 | Dehong | E98°58' | N24°43' | 914 | C74 |
| 319 | N24 | 2015.3.12 | Dehong | E98°58' | N24°43' | 914 | 98-62 |
| 320 | N25 | 2015.3.12 | Dehong | E98°58' | N24°43' | 914 | C116 |
| 321 | N26 | 2015.3.12 | Dehong | E98°58' | N24°43' | 914 | De Mai 3 |
| 322 | N27 | 2015.3.19 | Zhaotong | | | | Local wheat |
| 323 | N28 | 2015.3.19 | Zhaotong | | | | Qian Baode |
| 324 | N29 | 2015.3.19 | Zhaotong | | | | Khapli/8cc |
| 325 | N30 | 2015.3.19 | Zhaotong | | | | Kalyansna Yr2 |
| 326 | N31 | 2015.3.19 | Zhaotong | | | | Anti-citation 655 |
| 327 | N32 | 2015.3.19 | Zhaotong | | | | Aurora |
| 328 | N33 | 2015.3.16 | Lincang | E100°9'39" | N24°15'49" | 1829 | Linmai 6 |
| 329 | N34 | 2015.3.5 | Lincang | E99°14'10" | N23°59'2" | 1488 | Linmai 6 |
| 330 | N35 | 2015.3.5 | Lincang | E99°14'18" | N23°58'59" | 1493 | Linmai 6 |
| 331 | N36 | 2015.3.24 | Lincang | E100.613° | N23.573° | 1470 | Province J-50 |
| 332 | N37 | 2015.3.24 | Lincang | E100.613° | N23.573° | 1470 | Yunmai 11 |
| 333 | N38 | 2015.3.5 | Lincang | E99°14'40" | N24°0'6" | 1500 | Linmai 6 |
| 334 | N39 | 2015.3.24 | Lincang | E100°0'50" | N24°2'7" | 1580 | Linmai 6 |
| 335 | N40 | 2015.3.19 | Qujing | E103°51'2" | N25°36'46" | 1960 | Unknown variety |
| 336 | N41 | 2015.3.19 | Qujing | E103°51'2" | N25°36'46" | 1960 | Unknown variety |
| 337 | N42 | 2015.3.19 | Qujing | E103°51'2" | N25°36'46" | 1960 | Unknown variety |
| 338 | N43 | 2015.3.19 | Qujing | E103°51'2" | N25°36'46" | 1960 | Unknown variety |
| 339 | N44 | 2015.3.19 | Qujing | E103°51'2" | N25°36'46" | 1960 | Unknown variety |
| 340 | N45 | 2015.3.20 | Chuxiong | E101°34'11" | N25°2'14" | 1772 | Unknown variety |
| 341 | N46 | 2015.3.20 | Chuxiong | E101°34'11" | N25°2'14" | 1772 | Unknown variety |
| 342 | N47 | 2015.3.20 | Chuxiong | E101°34'11" | N25°2'14" | 1772 | Chuanmai 107 |

| Sl. No. | Isolate ID | Collection date | Location | Longitude | Latitude | Altitude (m) | Wheat cultivar |
|---|---|---|---|---|---|---|---|
| 343 | N48 | 2015.3.20 | Chuxiong | E101°34'11" | N25°2'14" | 1772 | Linmai 6 |
| 344 | N49 | 2015.3.20 | Chuxiong | E101°34'11" | N25°2'14" | 1772 | Yimai 1 |
| 345 | N50 | 2015.3.20 | Chuxiong | E101°34'11" | N25°2'14" | 1772 | Yunmai 39 |
| 346 | N51 | 2015.3.20 | Chuxiong | E101°34'11" | N25°2'14" | 1772 | Wenmai 16 |
| 347 | N52 | 2015.3.20 | Chuxiong | E101°34'11" | N25°2'14" | 1772 | Morocco |
| 348 | N53 | 2015.3.20 | Chuxiong | E101°34'11" | N25°2'14" | 1772 | Unknown variety |
| 349 | N54 | 2015.3.20 | Chuxiong | E101°34'11" | N25°2'14" | 1772 | Unknown variety |
| 350 | N55 | 2015.3.5 | Yuxi | E102°10'38" | N24°36'55" | 1620 | Yunmai 47 |
| 351 | N56 | 2015.3.5 | Yuxi | | | 1540 | 93-124 |
| 352 | N57 | 2015.3.15 | Yuxi | E102°29' | N24°19' | 1696 | Water source 11 |

**Table S2.** Haplotypes and their SNP loci of *Pst* population.

| | Locus, Consensus, Character | | | | | | | | | | | | | | | | | | | | | | | | | | | | | | | | | | | | | | | | | |
|---|---|---|---|---|---|---|---|---|---|---|---|---|---|---|---|---|---|---|---|---|---|---|---|---|---|---|---|---|---|---|---|---|---|---|---|---|---|---|---|---|---|---|
| | CDC | | | | | | | | | | | | | EF | | | | | HSP | | | | | | | MAP | | | | UBA | | | | | | UBC | | | | | | |
| | **C** | **G** | **G** | **T** | **T** | **G** | **G** | **G** | **G** | **A** | **G** | **A** | **A** | **T** | **A** | **T** | **C** | **G** | **C** | **C** | **G** | **G** | **C** | **T** | **G** | **G** | **A** | **C** | **G** | **C** | **G** | **A** | **C** | **A** | **A** | **C** | **A** | **T** | **G** | **T** | **C** | **T** |
| | **i** | **n** | **i** | **i** | **i** | **i** | **n** | **n** | **i** | **n** | **i** | **n** | **i** | **i** | **n** | **n** | **i** | **i** | **i** | **i** | **i** | **i** | **i** | **i** | **i** | **i** | **n** | **i** | **i** | **i** | **i** | **i** | **i** | **i** | **i** | **i** | **i** | **i** | **i** | **i** | **i** | **i** |
| H1 | . | . | . | . | . | . | . | . | . | . | . | . | G | . | . | . | . | . | T | . | . | . | . | . | . | A | . | T | T | . | . | . | . | . | . | . | . | . | . | . | . | C |
| H2 | T | . | . | . | . | . | . | T | . | . | . | . | G | . | . | . | . | . | T | A | . | C | . | . | . | . | . | . | . | . | . | . | . | . | . | T | . | . | . | G | . | C |
| H3 | T | . | . | A | . | . | . | . | . | . | . | . | . | . | . | . | . | . | T | A | C | . | A | C | . | . | . | . | T | . | . | . | . | . | . | . | . | C | . | . | . | . |
| H4 | . | C | A | A | . | A | . | . | A | T | . | T | . | . | . | . | . | . | T | A | C | . | A | C | . | . | . | . | . | . | . | . | . | . | . | T | G | . | A | G | G | C |
| H5 | . | . | A | A | . | A | A | A | A | . | . | . | . | . | . | . | . | . | T | A | C | . | A | C | . | . | . | . | . | . | . | . | . | . | . | T | G | . | A | G | G | C |
| H6 | . | . | . | A | . | . | . | . | . | . | T | . | . | C | . | . | T | C | . | . | . | C | . | . | . | . | . | T | T | T | T | . | T | . | . | . | . | C | . | . | . | . |
| H7 | . | . | . | A | . | . | . | . | . | . | . | . | . | C | . | . | T | C | . | . | . | C | . | . | . | . | . | . | . | . | . | . | . | . | . | . | . | . | . | . | . | C |
| H8 | . | . | . | A | . | . | . | . | . | . | . | . | . | . | . | . | . | . | . | . | . | . | . | . | . | . | . | . | . | . | . | . | . | . | . | . | . | . | . | . | . | . |
| H9 | . | . | . | A | . | . | . | . | . | . | . | . | . | . | . | A | . | . | T | A | C | . | . | . | . | . | . | . | . | . | . | G | T | G | C | . | . | C | . | . | . | C |
| H10 | . | . | . | A | . | . | . | . | . | . | . | . | . | . | . | . | . | . | T | . | . | . | . | . | . | . | . | . | . | . | . | G | T | G | C | . | . | . | . | . | . | C |
| H11 | . | . | . | A | . | . | . | . | . | . | . | . | . | . | . | . | . | . | T | . | C | . | . | . | . | . | . | . | . | . | . | G | T | G | C | . | . | C | . | . | . | C |
| H12 | . | . | . | A | . | . | . | . | . | . | . | . | . | . | . | . | . | . | T | A | . | . | . | . | . | . | . | . | . | . | . | . | . | . | . | . | . | C | . | . | . | . |
| H13 | . | . | . | A | . | . | . | . | . | . | . | . | . | . | . | . | . | . | T | A | . | . | . | . | . | . | . | . | . | . | . | . | . | . | . | . | . | . | . | G | . | C |
| H14 | . | . | . | A | . | . | . | . | . | . | . | . | . | . | . | . | . | . | T | A | . | . | . | . | . | . | . | . | . | . | . | . | . | . | . | . | . | C | . | . | . | C |
| H15 | . | . | . | A | . | . | . | . | . | . | . | . | . | . | . | . | . | . | T | A | C | . | A | C | . | . | . | . | . | . | . | . | . | . | . | . | . | C | . | . | . | . |
| H16 | . | . | . | A | . | . | . | . | . | . | . | . | . | . | . | . | . | . | T | A | C | . | A | . | . | . | . | . | . | . | . | . | . | . | . | T | G | . | A | G | G | C |
| H17 | . | . | . | A | . | . | . | . | . | . | . | . | . | . | . | . | . | . | T | A | C | . | A | C | . | A | . | T | . | . | . | . | . | . | . | . | . | C | . | . | . | . |
| H18 | . | . | . | A | . | . | . | . | . | . | . | . | . | . | . | . | . | . | T | A | C | . | A | C | . | . | . | T | T | . | . | . | . | . | . | . | . | C | . | . | . | . |
| H19 | . | . | . | A | . | . | . | . | . | . | . | . | . | . | . | . | . | . | T | A | C | . | A | C | . | . | . | T | T | . | . | . | . | . | . | T | G | . | A | G | G | C |
| H20 | . | . | . | A | . | . | . | . | . | . | . | . | . | . | . | . | . | . | T | A | C | . | A | C | . | . | . | . | T | . | . | . | . | . | . | . | . | C | . | . | . | . |
| H21 | . | . | . | A | . | . | . | . | . | . | . | . | . | . | . | . | . | . | T | A | C | . | A | C | . | . | . | . | T | . | . | . | . | . | . | T | G | . | A | G | G | C |
| H22 | . | . | . | A | . | . | . | . | . | . | . | . | . | . | . | . | . | . | T | A | C | . | A | C | . | . | . | . | . | . | . | . | . | . | . | T | . | C | . | . | . | . |
| H23 | . | . | . | A | . | . | . | . | . | . | . | . | . | . | . | . | . | . | T | A | C | . | A | C | . | . | . | . | . | . | . | . | . | . | . | T | G | . | A | G | G | C |
| H24 | . | . | . | A | . | . | . | . | . | . | . | . | . | . | . | . | . | . | T | A | C | . | A | C | . | . | . | . | . | . | . | . | . | . | . | T | . | . | . | G | . | C |
| H25 | . | . | . | A | . | . | . | . | . | . | . | . | . | . | . | . | . | . | T | A | C | . | A | C | . | . | . | . | . | . | . | . | . | . | . | . | . | . | . | . | . | . |
| H26 | . | . | . | A | . | . | . | . | . | . | . | . | . | . | . | . | . | . | T | A | C | . | A | C | . | . | . | . | . | . | . | . | . | . | . | . | . | . | . | G | . | C |
| H27 | . | . | . | A | . | . | . | . | . | . | . | . | . | . | . | . | . | . | T | A | C | . | . | C | . | . | . | . | . | . | . | . | . | . | . | . | . | . | . | . | . | . |
| H28 | . | . | . | A | . | . | . | . | . | . | . | . | . | . | . | . | . | . | T | A | C | . | . | . | . | . | . | . | . | . | . | . | . | . | . | . | . | . | . | . | . | . |
| H29 | . | . | . | A | . | . | . | . | . | . | . | . | . | . | . | . | . | . | T | A | C | . | . | . | . | . | . | . | . | . | . | G | T | G | C | . | . | . | . | G | . | C |

| | Locus, Consensus, Character | | | | | | | | | | | | | | | | | | | | | | | | | | | | | | | | | | | | | | | | | |
|---|---|---|---|---|---|---|---|---|---|---|---|---|---|---|---|---|---|---|---|---|---|---|---|---|---|---|---|---|---|---|---|---|---|---|---|---|---|---|---|---|---|---|
| | | | | | | | CDC | | | | | | | | | EF | | | | | | HSP | | | | | MAP | | | | | UBA | | | | | | | | UBC | | | |
| | **C** | **G** | **G** | **T** | **T** | **G** | **G** | **G** | **G** | **A** | **G** | **A** | **A** | **T** | **A** | **T** | **C** | **G** | **C** | **C** | **G** | **G** | **C** | **T** | **G** | **G** | **A** | **C** | **G** | **C** | **G** | **A** | **C** | **A** | **A** | **C** | **A** | **T** | **G** | **T** | **C** | **T** |
| | **i** | **n** | **i** | **i** | **i** | **i** | **n** | **n** | **i** | **n** | **i** | **n** | **i** | **i** | **n** | **n** | **i** | **i** | **i** | **i** | **i** | **i** | **i** | **i** | **i** | **i** | **n** | **i** | **i** | **i** | **i** | **i** | **i** | **i** | **i** | **i** | **i** | **i** | **i** | **i** | **i** | **i** |
| H30 | . | . | . | A | . | . | . | . | . | . | . | . | . | . | . | . | . | . | T | . | C | . | A | . | . | . | . | . | . | . | . | . | . | . | . | . | . | C | . | . | . | . |
| H31 | . | . | . | A | . | . | . | . | . | . | . | . | . | . | . | . | . | . | T | . | . | C | . | . | . | . | . | . | . | . | . | . | . | . | . | . | . | . | . | . | . | . |
| H32 | . | . | . | A | . | . | . | . | . | . | . | . | . | . | . | . | . | . | T | . | . | . | A | . | . | . | . | . | . | . | . | . | . | . | . | . | . | . | . | G | . | . |
| H33 | . | . | . | A | . | . | . | . | . | . | . | . | . | . | . | . | . | . | T | . | . | . | . | . | A | . | . | . | . | . | . | G | T | G | C | . | . | . | . | . | . | . |
| H34 | . | . | . | A | . | . | . | . | . | . | . | . | . | . | . | . | . | . | T | . | . | . | . | . | A | . | . | . | . | . | . | . | . | . | . | . | . | . | . | . | . | . |
| H35 | . | . | . | A | . | . | . | . | . | . | . | . | . | . | . | . | . | . | T | . | . | . | . | . | A | . | . | . | . | . | . | . | . | . | C | . | . | . | . | . | . | . |
| H36 | . | . | . | A | . | . | . | . | . | . | . | . | . | . | . | . | . | . | T | . | . | . | . | . | . | . | . | . | . | . | . | . | . | . | . | . | . | . | . | . | . | . |
| H37 | . | . | . | A | . | . | . | . | . | . | . | . | . | . | . | . | . | . | T | . | . | . | . | . | . | . | . | . | . | . | . | G | T | G | C | . | . | C | . | . | . | . |
| H38 | . | . | . | A | . | . | . | . | . | . | . | . | . | . | . | . | . | . | T | . | . | . | . | . | . | . | . | . | . | . | . | G | T | G | C | . | . | C | . | . | . | C |
| H39 | . | . | . | A | . | . | . | . | . | . | . | . | . | . | . | . | . | . | T | . | . | . | . | . | . | . | . | . | . | . | . | G | T | G | C | . | . | . | . | . | . | . |
| H40 | . | . | . | A | . | . | . | . | . | . | . | . | . | . | . | . | . | . | . | A | . | . | . | . | A | . | . | . | . | . | . | . | . | . | . | T | . | C | . | . | . | C |
| H41 | . | . | . | A | . | . | . | . | . | . | . | . | . | . | . | . | . | . | . | A | C | . | . | . | . | . | . | . | . | . | . | G | T | G | C | T | . | C | . | . | . | C |
| H42 | . | . | . | A | . | . | . | . | . | . | . | . | . | . | . | . | . | . | . | A | C | . | A | C | . | . | . | . | . | . | . | . | . | . | . | . | . | C | . | . | . | . |
| H43 | . | . | . | A | . | . | . | . | . | . | . | . | . | . | . | . | . | . | . | A | C | . | A | . | . | . | . | . | . | . | . | . | . | . | . | . | . | C | . | . | . | . |
| H44 | . | . | . | A | . | . | . | . | . | . | . | . | . | . | . | . | . | . | . | A | C | . | . | C | . | . | . | . | . | . | . | G | T | G | C | . | . | . | . | . | . | . |
| H45 | . | . | . | A | . | . | . | . | . | . | . | . | . | . | . | . | . | . | . | A | C | . | . | C | . | . | . | . | . | . | . | . | . | . | . | . | . | . | . | . | . | . |
| H46 | . | . | . | A | . | . | . | . | . | . | . | . | . | . | . | . | . | . | . | A | C | . | . | . | A | . | . | . | . | . | . | . | . | . | . | T | . | C | . | G | . | C |
| H47 | . | . | . | A | . | . | . | . | . | . | . | . | . | . | . | . | . | . | . | A | C | . | . | . | . | . | . | . | . | . | . | . | . | . | . | . | . | C | . | . | . | . |
| H48 | . | . | . | A | . | . | . | . | . | . | . | . | . | . | . | . | . | . | . | A | C | . | . | . | . | . | . | . | . | . | . | . | . | . | . | T | . | C | . | G | . | C |
| H49 | . | . | . | A | . | . | . | . | . | . | . | . | . | . | . | . | . | . | . | A | C | . | . | . | . | . | . | . | . | . | . | . | . | . | . | T | . | C | . | . | . | C |
| H50 | . | . | . | A | . | . | . | . | . | . | . | . | . | . | . | . | . | . | . | A | C | . | . | . | . | . | . | . | . | . | . | . | . | . | . | T | . | C | . | . | . | . |
| H51 | . | . | . | A | . | . | . | . | . | . | . | . | . | . | . | . | . | . | . | A | C | . | . | . | . | . | . | . | . | . | . | . | . | . | . | . | . | . | . | . | . | . |
| H52 | . | . | . | A | . | . | . | . | . | . | . | . | . | . | . | . | . | . | . | A | C | . | . | . | . | . | . | . | . | . | . | . | . | . | . | . | . | . | A | G | . | . |
| H53 | . | . | . | A | . | . | . | . | . | . | . | . | . | . | . | . | . | . | . | A | C | . | . | . | . | . | . | . | . | . | . | . | . | . | . | . | . | . | . | . | . | C |
| H54 | . | . | . | A | . | . | . | . | . | . | . | . | . | . | . | . | . | . | . | A | C | . | . | . | . | . | . | . | . | . | . | G | T | G | C | . | . | . | . | . | . | . |
| H55 | . | . | . | A | . | . | . | . | . | . | . | . | . | . | . | . | . | . | . | A | C | . | . | . | . | . | . | . | . | . | . | G | T | G | C | . | . | C | . | . | . | . |
| H56 | . | . | . | A | . | . | . | . | . | . | . | . | . | . | . | . | . | . | . | A | . | . | . | . | . | . | . | . | . | . | . | G | T | G | C | T | . | C | . | . | . | C |
| H57 | . | . | . | A | . | . | . | . | . | . | . | . | . | . | . | . | . | . | . | A | . | . | . | . | A | . | . | . | . | . | . | G | T | G | C | . | . | . | . | G | . | . |
| H58 | . | . | . | A | . | . | . | . | . | . | . | . | . | . | . | . | . | . | . | A | . | . | . | . | A | . | . | . | . | . | . | G | T | G | C | . | . | C | A | . | . | . |
| H59 | . | . | . | A | . | . | . | . | . | . | . | . | . | . | . | . | . | . | . | . | . | C | . | . | . | . | . | . | . | . | . | . | . | . | . | . | . | C | . | . | . | C |

| | Locus, Consensus, Character | | | | | | | | | | | | | | | | | | | | | | | | | | | | | | | | | | | | | | | | |
|---|---|---|---|---|---|---|---|---|---|---|---|---|---|---|---|---|---|---|---|---|---|---|---|---|---|---|---|---|---|---|---|---|---|---|---|---|---|---|---|---|---|
| | | | | | | **CDC** | | | | | | | | | **EF** | | | | | | **HSP** | | | | | **MAP** | | | | | | **UBA** | | | | | | **UBC** | | | |
| | **C** | **G** | **G** | **T** | **T** | **G** | **G** | **G** | **G** | **A** | **G** | **A** | **A** | **T** | **A** | **T** | **C** | **G** | **C** | **C** | **G** | **G** | **C** | **T** | **G** | **G** | **A** | **C** | **G** | **C** | **G** | **A** | **C** | **A** | **A** | **C** | **A** | **T** | **G** | **T** | **C** | **T** |
| | **i** | **n** | **i** | **i** | **i** | **i** | **n** | **n** | **i** | **n** | **i** | **n** | **i** | **i** | **n** | **n** | **i** | **i** | **i** | **i** | **i** | **i** | **i** | **i** | **i** | **i** | **n** | **i** | **i** | **i** | **i** | **i** | **i** | **i** | **i** | **i** | **i** | **i** | **i** | **i** | **i** | **i** |
| H60 | . | . | . | A | . | . | . | . | . | . | . | . | . | . | . | . | . | . | . | . | . | C | . | . | A | . | . | . | . | . | . | . | . | . | . | T | . | . | . | . | . | C |
| H61 | . | . | . | A | . | . | . | . | . | . | . | . | . | . | . | . | . | . | . | . | . | C | . | . | A | . | . | . | . | . | . | . | . | . | . | . | . | C | . | . | . | C |
| H62 | . | . | . | A | . | . | . | . | . | . | . | . | . | . | . | . | . | . | . | . | . | C | . | . | A | . | . | . | . | . | . | . | . | . | . | T | G | . | . | . | . | C |
| H63 | . | . | . | A | . | . | . | . | . | . | . | . | . | . | . | . | . | . | . | . | . | C | . | . | . | . | . | . | . | . | . | . | . | . | . | T | . | C | . | . | . | C |
| H64 | . | . | . | A | . | . | . | . | . | . | . | . | . | . | . | . | . | . | . | . | . | C | . | . | . | . | . | . | . | . | . | G | T | G | C | | . | . | . | . | . | C |
| H65 | . | . | . | A | . | . | . | . | . | . | . | . | . | . | . | . | . | . | . | . | . | C | . | . | . | . | . | . | . | . | . | G | T | G | C | T | . | C | . | . | . | C |
| H66 | . | . | . | A | . | . | . | . | . | . | . | . | . | . | . | . | . | . | . | . | . | C | . | . | . | . | . | . | . | . | . | G | T | G | C | . | . | C | . | . | . | C |
| H67 | . | . | . | A | . | . | . | . | . | . | . | . | . | . | . | . | . | . | . | . | . | C | . | . | . | . | . | . | . | . | . | G | T | G | C | . | . | . | . | G | . | . |
| H68 | . | . | . | A | . | . | . | . | . | . | . | . | . | . | . | . | . | . | . | . | . | C | . | . | . | . | . | . | . | . | . | G | T | G | C | . | . | . | . | . | . | . |
| H69 | . | . | . | A | . | . | . | . | . | . | . | . | . | . | . | . | . | . | . | . | . | C | . | . | . | . | . | . | . | . | . | . | . | G | . | T | . | C | . | . | . | C |
| H70 | . | . | . | A | . | . | . | . | . | . | . | . | . | . | . | . | . | . | . | . | . | C | . | . | . | . | . | . | . | . | . | . | . | . | . | T | . | . | . | . | . | C |
| H71 | . | . | . | A | . | . | . | . | . | . | . | . | . | . | . | . | . | . | . | . | . | C | . | . | . | . | . | . | . | . | . | . | . | . | . | T | . | . | . | G | . | C |
| H72 | . | . | . | A | . | . | . | . | . | . | . | . | . | . | . | . | . | . | . | . | . | C | . | . | . | . | . | . | . | . | . | . | . | . | . | . | . | . | . | . | . | . |
| H73 | . | . | . | A | . | . | . | . | . | . | . | . | . | . | . | . | . | . | . | . | . | C | . | . | . | . | . | . | . | . | . | . | . | . | . | . | . | . | . | G | . | C |
| H74 | . | . | . | A | . | . | . | . | . | . | . | . | . | . | . | . | . | . | . | . | . | . | . | . | A | . | . | . | . | . | . | . | . | . | . | . | . | C | . | . | . | . |
| H75 | . | . | . | A | . | . | . | . | . | . | . | . | . | . | . | . | . | . | . | . | . | . | . | . | A | . | . | . | . | . | . | . | . | . | . | . | . | . | . | . | . | C |
| H76 | . | . | . | A | . | . | . | . | . | . | . | . | . | . | . | . | . | . | . | . | . | . | . | . | . | . | . | . | . | . | . | G | T | G | C | . | . | . | . | . | . | C |
| H77 | . | . | . | A | . | . | . | . | . | . | . | . | . | . | . | . | . | . | . | . | . | . | . | . | . | . | . | . | . | . | . | G | | G | C | . | . | . | . | . | . | . |
| H78 | . | . | . | A | . | . | . | . | . | . | . | . | . | . | . | . | . | . | . | . | . | . | . | . | . | . | . | . | . | . | . | G | T | G | C | T | . | . | . | . | . | C |
| H79 | . | . | . | A | . | . | . | . | . | . | . | . | . | . | . | . | . | . | . | . | . | . | . | . | . | . | . | . | . | . | . | G | T | G | C | T | . | C | . | . | . | C |
| H80 | . | . | . | A | . | . | . | . | . | . | . | . | . | . | . | . | . | . | . | . | . | . | . | . | . | . | . | . | . | . | . | G | T | G | C | T | . | C | . | . | . | . |
| H81 | . | . | . | A | . | . | . | . | . | . | . | . | . | . | . | . | . | . | . | . | . | . | . | . | . | . | . | . | . | . | . | G | T | G | C | T | . | | . | . | . | . |
| H82 | . | . | . | A | . | . | . | . | . | . | . | . | . | . | . | . | . | . | . | . | . | . | . | . | . | . | . | . | . | . | . | G | T | G | C | . | . | C | . | . | . | C |
| H83 | . | . | . | A | . | . | . | . | . | . | . | . | . | . | . | . | . | . | . | . | . | . | . | . | . | . | . | . | . | . | . | G | T | G | C | . | . | C | . | . | . | . |
| H84 | . | . | . | A | . | . | . | . | . | . | . | . | . | . | . | . | . | . | . | . | . | . | . | . | . | . | . | . | . | . | . | G | T | G | C | . | . | . | . | G | . | C |
| H85 | . | . | . | A | . | . | . | . | . | . | . | . | . | . | . | . | . | . | . | . | . | . | . | . | . | . | . | . | . | . | . | G | T | G | C | . | . | . | . | G | . | . |
| H86 | . | . | . | A | . | . | . | . | . | . | . | . | . | . | . | . | . | . | . | . | . | . | . | . | . | . | . | . | . | . | . | G | T | G | C | . | . | . | . | . | . | . |
| H87 | . | . | . | A | . | . | . | . | . | . | . | . | . | . | . | . | . | . | . | . | . | . | . | . | . | . | . | . | . | . | . | . | . | . | . | T | . | C | . | . | . | C |
| H88 | . | . | . | A | . | . | . | . | . | . | . | . | . | . | . | . | . | . | . | . | . | . | . | . | . | . | . | . | . | . | . | . | . | . | . | T | . | . | . | . | . | C |
| H89 | . | . | . | A | . | . | . | . | . | . | . | . | . | . | . | . | . | . | . | . | . | . | . | . | . | . | . | . | . | . | . | . | . | . | . | T | . | . | . | . | . | . |

| | Locus, Consensus, Character | | | | | | | | | | | | | | | | | | | | | | | | | | | | | | | | | | | | | | | | | |
|---|---|---|---|---|---|---|---|---|---|---|---|---|---|---|---|---|---|---|---|---|---|---|---|---|---|---|---|---|---|---|---|---|---|---|---|---|---|---|---|---|---|---|
| | | | | | | | CDC | | | | | | | | | EF | | | | | | HSP | | | | | MAP | | | | | | UBA | | | | | | UBC | | | |
| | **C** | **G** | **G** | **T** | **T** | **G** | **G** | **G** | **G** | **A** | **G** | **A** | **A** | **T** | **A** | **T** | **C** | **G** | **C** | **C** | **G** | **G** | **C** | **T** | **G** | **G** | **A** | **C** | **G** | **C** | **G** | **A** | **C** | **A** | **A** | **C** | **A** | **T** | **G** | **T** | **C** | **T** |
| | **i** | **n** | **i** | **i** | **i** | **i** | **n** | **n** | **i** | **n** | **i** | **n** | **i** | **i** | **n** | **n** | **i** | **i** | **i** | **i** | **i** | **i** | **i** | **i** | **i** | **i** | **n** | **i** | **i** | **i** | **i** | **i** | **i** | **i** | **i** | **i** | **i** | **i** | **i** | **i** | **i** | **i** |
| H90 | . | . | . | A | . | . | . | . | . | . | . | . | . | . | . | . | . | . | . | . | . | . | . | . | . | . | . | . | . | . | . | . | . | . | . | . | . | C | . | G | . | . |
| H91 | . | . | . | A | . | . | . | . | . | . | . | . | . | . | . | . | . | . | . | . | . | . | . | . | . | . | . | . | . | . | . | . | . | . | . | . | . | C | . | . | . | . |
| H92 | . | . | . | A | . | . | . | . | . | . | . | . | . | . | . | . | . | . | . | . | . | . | . | . | . | . | . | . | . | . | . | . | . | . | . | . | . | C | . | . | . | C |
| H93 | . | . | . | A | . | . | . | . | . | . | . | . | . | . | . | . | . | . | . | . | . | . | . | . | . | . | . | . | . | . | . | . | . | . | . | . | . | . | . | G | . | . |
| H94 | . | . | . | A | . | . | . | . | . | . | . | . | . | . | . | . | . | . | . | . | . | . | . | . | . | . | . | . | . | . | . | . | . | . | . | . | . | . | . | . | . | C |
| H95 | . | . | . | A | . | . | . | . | . | . | . | . | . | C | . | . | . | . | T | A | C | | . | . | . | . | . | . | . | . | . | . | . | . | . | . | . | . | . | . | . | C |
| H96 | . | . | . | A | . | . | . | . | . | . | . | . | . | C | . | . | . | . | T | . | . | C | . | . | . | . | . | . | . | . | . | G | T | G | C | . | . | . | . | G | . | C |
| H97 | . | . | . | A | . | . | . | . | . | . | . | . | . | C | . | . | T | . | T | A | . | . | . | . | . | . | . | . | . | . | . | . | . | . | . | T | . | . | . | . | . | C |
| H98 | . | . | . | A | . | . | . | . | . | . | . | . | . | C | . | . | T | . | . | A | C | . | . | . | . | . | . | . | . | . | . | . | . | . | . | . | . | . | . | . | . | . |
| H99 | . | . | . | A | . | . | . | . | . | . | . | . | . | C | . | . | T | C | T | A | C | . | A | . | . | . | . | . | . | . | . | . | . | . | . | . | . | C | . | . | . | . |
| H100 | . | . | . | A | . | . | . | . | . | . | . | . | . | C | . | . | T | C | T | . | . | . | . | . | . | . | . | . | . | . | . | G | T | G | C | . | . | . | . | . | . | . |
| H101 | . | . | . | A | . | . | . | . | . | . | . | . | . | C | . | . | T | C | . | . | . | C | . | . | . | . | . | . | . | . | . | G | T | G | C | . | . | . | . | . | . | C |
| H102 | . | . | . | A | . | . | . | . | . | . | . | . | . | C | . | . | T | C | . | . | . | C | . | . | . | . | . | . | . | . | . | G | T | G | C | . | . | . | . | . | . | . |
| H103 | . | . | . | A | . | . | . | . | . | . | T | . | . | C | G | . | T | C | . | A | . | . | . | . | . | . | . | T | T | T | T | . | T | . | . | . | . | C | . | . | . | . |
| H104 | . | . | . | . | C | . | . | . | . | . | . | . | . | . | . | . | . | . | T | A | C | . | A | C | . | A | . | T | T | . | . | . | . | . | . | T | G | . | A | G | G | C |
| H105 | . | . | . | . | C | . | . | . | . | . | . | . | . | . | . | . | . | . | T | A | C | . | A | C | . | | . | T | T | . | . | . | . | . | . | T | G | . | A | G | G | C |
| H106 | . | . | . | . | . | . | . | . | . | . | . | . | . | . | . | . | . | . | T | . | . | C | . | . | . | A | . | T | T | . | . | . | . | . | . | . | . | . | . | . | . | C |
| H107 | . | . | . | . | . | . | . | . | . | . | . | . | . | C | . | . | . | . | T | . | . | . | . | . | . | A | . | T | T | . | . | . | . | . | . | . | . | . | . | . | . | . |
| H108 | . | . | . | . | . | . | . | . | . | . | . | . | . | C | . | . | T | . | . | . | . | . | . | . | . | A | . | T | T | . | . | . | . | . | . | . | . | . | . | . | . | . |
| H109 | . | . | . | . | . | . | . | . | . | . | . | . | . | C | . | . | T | C | . | . | . | . | . | . | . | A | . | T | T | . | . | . | . | . | . | . | . | . | . | G | . | . |
| H110 | . | . | . | . | . | . | . | . | . | . | . | . | . | C | . | . | T | C | T | . | . | C | A | . | . | . | . | T | T | . | . | . | . | . | . | . | . | . | . | . | . | . |
| H111 | . | . | . | . | . | . | . | . | . | . | . | . | . | C | . | . | T | C | T | A | . | . | . | . | . | . | . | | T | . | . | . | . | . | . | . | . | . | . | G | . | C |
| H112 | . | . | . | . | . | . | . | . | . | . | . | . | . | C | . | . | T | C | T | . | . | . | . | . | . | . | . | T | T | . | . | . | . | . | . | . | . | . | . | . | . | . |
| H113 | . | . | . | . | . | . | . | . | . | . | . | . | . | C | . | . | T | C | T | . | . | . | . | . | . | . | . | . | . | . | . | . | . | . | . | . | . | . | . | . | . | . |
| H114 | . | . | . | . | . | . | . | . | . | . | . | . | . | C | . | . | T | C | . | . | . | C | . | . | . | A | . | T | T | . | . | . | . | . | . | . | . | . | . | . | . | . |
| H115 | . | . | . | . | . | . | . | . | . | . | . | . | . | C | . | . | T | C | . | . | . | C | . | . | . | . | . | T | T | . | . | . | . | . | . | . | . | . | . | . | . | . |
| H116 | . | . | . | . | . | . | . | . | . | . | . | . | . | C | . | . | T | C | . | . | . | . | . | . | . | . | . | T | T | . | . | . | . | . | . | . | . | . | . | . | . | . |
| H117 | . | . | . | . | . | . | . | . | . | . | . | . | . | C | . | . | T | C | . | . | . | . | . | . | . | . | . | . | T | . | . | . | . | . | . | . | . | . | . | . | . | . |
| H118 | . | . | . | . | . | . | . | . | . | . | . | . | . | C | . | . | T | C | . | . | . | . | . | . | . | . | . | T | . | . | . | . | . | . | . | . | . | . | . | . | . | . |
| H119 | . | . | . | . | . | . | . | . | . | . | . | . | . | C | . | . | T | C | . | . | . | . | . | . | . | . | . | T | T | . | . | . | . | . | . | . | . | C | . | G | . | . |

| | | | | | | | | | | | | | | | | | | | | | | | | | | | | | | | | | | | | | | | | | | |
|---|---|---|---|---|---|---|---|---|---|---|---|---|---|---|---|---|---|---|---|---|---|---|---|---|---|---|---|---|---|---|---|---|---|---|---|---|---|---|---|---|---|---|
| | **Locus, Consensus, Character** | | | | | | | | | | | | | | | | | | | | | | | | | | | | | | | | | | | | | | | | | |
| | | | | | | | **CDC** | | | | | | | | | **EF** | | | | | | **HSP** | | | | | **MAP** | | | | | **UBA** | | | | | | | **UBC** | | | |
| | **C** | **G** | **G** | **T** | **T** | **G** | **G** | **G** | **G** | **A** | **G** | **A** | **A** | **T** | **A** | **T** | **C** | **G** | **C** | **C** | **G** | **G** | **C** | **T** | **G** | **G** | **A** | **C** | **G** | **C** | **G** | **A** | **C** | **A** | **A** | **C** | **A** | **T** | **G** | **T** | **C** | **T** |
| | **i** | **n** | **i** | **i** | **i** | **i** | **n** | **n** | **i** | **n** | **i** | **n** | **i** | **i** | **n** | **n** | **i** | **i** | **i** | **i** | **i** | **i** | **i** | **i** | **i** | **i** | **n** | **i** | **i** | **i** | **i** | **i** | **i** | **i** | **i** | **i** | **i** | **i** | **i** | **i** | **i** | **i** |
| H120 | . | . | . | . | . | . | . | . | . | . | . | . | . | C | . | . | T | C | . | . | . | . | . | . | . | . | . | T | T | . | . | . | . | . | . | . | . | . | . | . | . | C |
| H121 | . | . | . | . | . | . | . | . | . | . | . | . | . | C | . | . | T | C | . | . | . | . | . | . | . | A | . | T | T | . | . | . | . | . | . | . | . | . | . | . | . | . |
| H122 | . | . | . | . | . | . | . | . | . | . | . | . | . | C | . | . | T | . | . | . | . | C | . | . | A | . | . | T | T | . | . | . | . | . | . | . | . | C | . | G | . | . |
| H123 | . | . | . | . | . | . | . | . | . | . | . | . | . | C | . | . | T | . | . | . | . | C | . | . | . | . | . | T | T | . | . | . | . | . | . | . | . | . | . | . | . | C |
| H124 | . | . | . | . | . | . | . | . | . | . | . | . | . | C | . | . | T | . | . | . | . | C | . | . | . | . | . | T | T | . | . | . | . | . | . | . | . | . | . | . | . | . |
| H125 | . | . | . | . | . | . | . | . | . | . | . | . | . | C | . | . | . | . | . | . | . | . | . | . | . | A | . | T | T | . | . | . | . | . | . | . | . | . | . | . | . | . |
| H126 | . | . | . | . | . | . | . | . | . | . | . | . | . | C | . | . | . | . | . | . | . | C | . | . | . | . | . | T | T | . | . | . | . | . | . | . | . | . | . | . | . | . |
| H127 | . | . | . | . | . | . | . | . | . | . | . | . | . | C | . | . | . | . | . | . | . | . | . | . | . | . | . | T | T | . | . | . | . | . | . | . | . | . | . | . | . | . |
| H128 | . | . | . | . | . | . | . | . | . | . | . | . | . | C | . | . | . | . | . | . | . | . | . | . | . | . | . | . | T | . | . | . | . | . | . | . | . | C | . | . | . | . |
| H129 | . | . | . | . | . | . | . | . | . | . | . | . | . | C | . | . | . | . | . | . | . | . | . | . | . | . | . | T | . | . | . | . | . | . | . | . | . | . | . | . | . | . |
| H130 | . | . | . | . | . | . | . | . | . | . | . | . | . | . | . | . | . | . | . | . | . | . | . | . | . | A | . | . | T | . | . | . | . | . | . | . | G | . | A | . | . | C |
| H131 | . | . | . | . | . | . | . | . | . | . | . | . | . | . | . | . | . | . | . | A | C | . | A | C | . | . | . | . | . | . | . | . | . | . | . | . | . | C | . | . | . | . |
| H132 | . | . | . | . | . | . | . | . | . | . | . | . | . | . | . | . | . | . | . | A | . | . | A | . | . | . | . | . | T | . | . | . | . | . | . | . | . | . | . | G | . | C |
| H133 | . | . | . | . | . | . | . | . | . | . | . | . | . | . | . | . | . | . | . | A | . | . | . | . | . | . | . | T | T | . | . | . | . | . | . | . | . | . | . | G | . | C |
| H134 | . | . | . | . | . | . | . | . | . | . | . | . | . | . | . | . | . | . | . | A | C | . | . | . | . | . | . | . | . | . | . | . | . | . | . | T | . | C | . | . | . | . |
| H135 | . | . | . | . | . | . | . | . | . | . | . | . | . | . | . | . | . | . | . | A | C | . | . | . | . | . | . | . | . | . | . | . | . | . | . | . | . | . | . | G | . | C |
| H136 | . | . | . | . | . | . | . | . | . | . | . | . | . | . | . | . | . | . | . | A | C | . | . | . | . | . | . | . | . | . | . | . | . | . | . | T | . | C | . | G | . | . |
| H137 | . | . | . | . | . | . | . | . | . | . | . | . | . | . | . | . | . | . | . | A | C | . | A | . | . | . | . | T | T | . | . | . | . | . | . | . | . | . | . | G | . | C |
| H138 | . | . | . | . | . | . | . | . | . | . | . | . | . | . | . | . | . | . | . | . | C | C | . | . | . | A | . | T | T | . | . | . | . | . | . | . | . | C | . | G | . | . |
| H139 | | . | . | . | . | . | . | . | . | . | . | . | . | . | . | . | . | . | . | . | C | C | . | . | . | A | . | T | T | . | . | . | . | . | . | . | . | . | . | . | . | C |
| H140 | . | . | . | . | . | . | . | . | . | . | . | . | . | . | . | . | . | . | . | . | . | C | . | . | . | . | . | T | T | . | . | . | . | . | . | . | . | . | . | . | . | C |
| H141 | . | . | . | . | . | . | . | . | . | . | . | . | . | . | . | . | . | . | . | . | . | C | . | . | . | A | . | T | T | . | . | . | . | . | . | . | . | . | . | . | . | . |
| H142 | . | . | . | . | . | . | . | . | . | . | . | . | . | . | . | . | . | . | . | . | . | C | . | . | . | A | . | . | T | . | . | . | . | . | . | . | . | . | . | . | . | . |
| H143 | . | . | . | . | . | . | . | . | . | . | . | . | . | . | . | . | . | . | . | . | . | C | . | . | . | . | . | . | T | . | . | . | . | . | . | . | . | . | . | . | . | C |
| H144 | . | . | . | . | . | . | . | . | . | . | . | . | . | . | . | . | . | . | . | . | . | C | . | . | . | . | . | . | . | . | . | . | . | . | . | . | . | . | . | . | . | C |
| H145 | . | . | . | . | . | . | . | . | . | . | . | . | . | . | . | . | . | . | . | . | . | C | . | . | . | . | . | . | . | . | . | . | . | . | . | T | . | C | . | . | . | C |
| H146 | . | . | . | . | . | . | . | . | . | . | . | . | . | . | . | . | . | . | . | . | . | C | . | . | . | . | . | T | T | . | . | . | . | . | . | . | . | . | . | . | . | . |
| H147 | . | . | . | . | . | . | . | . | . | . | . | . | . | . | . | . | . | . | . | . | . | . | . | . | . | . | . | T | T | . | . | . | . | . | . | . | . | C | . | . | . | . |
| H148 | . | . | . | . | . | . | . | . | . | . | . | . | . | . | . | . | . | . | . | . | . | . | . | . | . | . | . | . | . | . | . | . | . | . | . | . | . | . | . | . | . | . |
| H149 | . | . | . | . | . | . | . | . | . | . | . | . | . | . | . | . | . | . | . | . | . | . | . | . | . | . | . | T | T | . | . | . | . | . | . | . | . | . | . | . | . | . |

| | | | | | | | | | | | | | | | | | | | | | Locus, Consensus, Character | | | | | | | | | | | | | | | | | | | | | |
|---|---|---|---|---|---|---|---|---|---|---|---|---|---|---|---|---|---|---|---|---|---|---|---|---|---|---|---|---|---|---|---|---|---|---|---|---|---|---|---|---|---|
| | | | | | | | **CDC** | | | | | | | | | **EF** | | | | | | **HSP** | | | | | **MAP** | | | | | **UBA** | | | | | | | **UBC** | | | |
| | **C** | **G** | **G** | **T** | **T** | **G** | **G** | **G** | **G** | **A** | **G** | **A** | **A** | **T** | **A** | **T** | **C** | **G** | **C** | **C** | **G** | **G** | **C** | **T** | **G** | **G** | **A** | **C** | **G** | **C** | **G** | **A** | **C** | **A** | **A** | **C** | **A** | **T** | **G** | **T** | **C** | **T** |
| | **i** | **n** | **i** | **i** | **i** | **i** | **n** | **n** | **i** | **n** | **i** | **n** | **i** | **i** | **n** | **n** | **i** | **i** | **i** | **i** | **i** | **i** | **i** | **i** | **i** | **i** | **n** | **i** | **i** | **i** | **i** | **i** | **i** | **i** | **i** | **i** | **i** | **i** | **i** | **i** | **i** | **i** |
| H150 | . | . | . | . | . | . | . | . | . | . | . | . | . | . | . | . | . | . | . | . | . | . | . | . | . | . | . | T | T | . | . | . | . | . | . | . | . | C | . | G | . | . |
| H151 | . | . | . | . | . | . | . | . | . | . | . | . | . | . | . | . | . | . | . | . | . | . | . | . | . | A | . | T | T | . | . | . | . | . | . | . | . | . | A | . | . | C |
| H152 | . | . | . | . | . | . | . | . | . | . | . | . | . | . | . | . | . | . | . | . | . | . | . | . | . | A | . | T | T | . | . | . | . | . | . | . | . | C | . | . | . | . |
| H153 | . | . | . | . | . | . | . | . | . | . | . | . | . | . | . | . | . | . | . | . | . | . | . | . | . | A | . | T | T | . | . | . | . | . | . | . | . | . | . | . | . | C |
| H154 | . | . | . | . | . | . | . | . | . | . | . | . | . | . | . | . | . | . | . | . | . | . | . | . | . | A | . | T | T | . | . | . | . | . | . | . | . | . | . | G | | C |
| H155 | . | . | . | . | . | . | . | . | . | . | . | . | . | . | . | . | . | . | . | . | . | . | . | . | . | A | . | T | T | . | . | . | . | . | . | . | . | . | . | . | . | . |
| H156 | . | . | . | . | . | . | . | . | . | . | . | . | . | . | . | . | . | . | . | . | . | . | . | . | . | A | . | . | T | . | . | . | . | . | . | T | . | . | . | . | . | . |
| H157 | . | . | . | . | . | . | . | . | . | . | . | . | . | . | . | . | . | . | T | A | . | . | . | . | . | . | . | . | . | . | . | . | . | . | . | . | . | . | . | . | . | C |
| H158 | . | . | . | . | . | . | . | . | . | . | . | . | . | . | . | . | . | . | T | A | C | . | A | C | . | . | . | . | . | . | . | . | . | . | . | . | . | C | . | . | . | . |
| H159 | . | . | . | . | . | . | . | . | . | . | . | . | . | . | . | . | . | . | T | A | C | . | . | C | . | . | . | . | . | . | . | . | . | . | . | . | . | . | . | G | . | . |
| H160 | . | . | . | . | . | . | . | . | . | . | . | . | . | . | . | . | . | . | T | A | C | . | . | C | . | T | . | . | T | . | . | . | . | . | . | . | . | . | . | . | . | . |
| H161 | . | . | . | . | . | . | . | . | . | . | . | . | . | . | . | . | . | . | T | A | C | . | A | . | . | . | . | T | T | . | . | . | . | . | . | T | . | . | A | G | G | C |
| H162 | . | . | . | . | . | . | . | . | . | . | . | . | . | . | . | . | . | . | T | A | C | . | A | C | . | A | . | T | T | . | . | . | . | . | . | T | G | . | A | G | G | C |
| H163 | . | . | . | . | . | . | . | . | . | . | . | . | . | . | . | . | . | . | T | A | C | . | A | C | . | . | G | . | T | . | . | . | . | . | . | T | G | . | A | G | G | C |
| H164 | . | . | . | . | . | . | . | . | . | . | . | . | . | . | . | . | . | . | T | A | C | . | A | C | . | . | . | . | . | . | . | . | . | . | . | T | G | . | A | G | G | C |
| H165 | . | . | . | . | . | . | . | . | . | . | . | . | . | . | . | . | . | . | T | A | C | . | A | C | . | . | . | . | . | . | . | . | . | . | . | T | . | . | A | G | G | C |
| H166 | . | . | . | . | . | . | . | . | . | . | . | . | . | . | . | . | . | . | T | A | . | . | A | . | . | . | . | . | . | . | . | . | . | . | . | . | . | . | . | . | . | . |
| H167 | . | . | . | . | . | . | . | . | . | . | . | . | . | . | . | . | . | . | T | A | . | . | A | C | . | . | . | T | T | . | . | . | . | . | . | . | . | . | . | G | . | . |
| H168 | . | . | . | . | . | . | . | . | . | . | . | . | . | . | . | . | . | . | T | . | C | . | A | C | . | A | . | T | T | . | . | . | . | . | . | . | . | . | . | G | . | C |
| H169 | . | . | . | . | . | . | . | . | . | . | . | . | . | . | . | . | . | . | T | . | . | . | . | . | . | A | . | T | T | . | . | . | . | . | . | . | . | C | . | . | . | . |
| H170 | . | . | . | . | . | . | . | . | . | . | . | . | . | . | . | . | . | . | T | . | . | . | A | . | . | . | . | T | T | . | . | . | . | . | . | . | . | . | . | . | . | C |
| H171 | . | . | . | . | . | . | . | . | . | . | . | . | . | . | . | . | . | . | T | . | . | . | . | . | . | . | . | T | T | . | . | . | . | . | . | . | . | . | . | G | . | C |
| H172 | . | . | . | . | . | . | . | . | . | . | . | . | . | . | . | . | . | . | T | . | . | . | . | . | . | . | . | . | T | . | . | . | . | . | . | . | . | . | . | . | . | . |
| H173 | . | . | . | . | . | . | . | . | . | . | . | . | . | . | . | . | . | . | T | . | . | . | . | . | . | . | . | T | T | . | . | . | . | . | . | . | . | . | . | . | . | . |
| H174 | . | . | . | . | . | . | . | . | . | . | . | . | . | . | . | . | . | . | T | . | . | . | . | . | . | A | . | . | T | . | . | . | . | . | . | . | . | C | . | . | . | C |
| H175 | . | . | . | . | . | . | . | . | . | . | . | . | . | . | . | . | . | . | T | . | . | . | . | . | . | A | . | . | T | . | . | . | . | . | . | . | . | C | . | G | . | . |
| H176 | . | . | . | . | . | . | . | . | . | . | . | . | . | . | . | . | . | . | T | . | . | . | . | . | . | A | . | T | T | . | . | . | . | . | . | . | . | . | . | . | . | . |
| H177 | . | . | . | . | . | . | . | . | . | . | . | . | . | . | . | . | . | . | T | . | . | . | . | . | . | A | . | T | T | . | . | . | . | . | . | . | . | . | A | . | . | . |
| H178 | . | . | . | . | . | . | . | . | . | . | . | . | . | . | . | . | . | . | T | . | . | C | . | . | . | . | . | T | T | . | . | . | . | . | . | . | . | . | . | . | . | . |
| H179 | . | . | . | . | . | . | . | . | . | . | . | . | G | C | . | . | . | . | . | . | . | C | . | . | . | . | . | T | T | . | . | . | . | . | . | . | . | C | . | G | . | . |

| | Locus, Consensus, Character | | | | | | | | | | | | | | | | | | | | | | | | | | | | | | | | | | | | | | | | | |
|---|---|---|---|---|---|---|---|---|---|---|---|---|---|---|---|---|---|---|---|---|---|---|---|---|---|---|---|---|---|---|---|---|---|---|---|---|---|---|---|---|---|---|
| | **CDC** | | | | | | | | | | | | | **EF** | | | | | **HSP** | | | | | | | **MAP** | | | | **UBA** | | | | | | **UBC** | | | | | | |
| | **C** | **G** | **G** | **T** | **T** | **G** | **G** | **G** | **G** | **A** | **G** | **A** | **A** | **T** | **A** | **T** | **C** | **G** | **C** | **C** | **G** | **G** | **C** | **T** | **G** | **G** | **A** | **C** | **G** | **C** | **G** | **A** | **C** | **A** | **A** | **C** | **A** | **T** | **G** | **T** | **C** | **T** |
| | **i** | **n** | **i** | **i** | **i** | **i** | **n** | **n** | **i** | **n** | **i** | **n** | **i** | **i** | **n** | **n** | **i** | **i** | **i** | **i** | **i** | **i** | **i** | **i** | **i** | **i** | **n** | **i** | **i** | **i** | **i** | **i** | **i** | **i** | **i** | **i** | **i** | **i** | **i** | **i** | **i** | **i** |
| H180 | . | . | . | . | . | . | . | . | . | . | . | . | G | C | . | . | T | C | . | . | . | . | . | . | . | . | . | T | T | . | . | . | . | . | . | . | . | C | . | . | . | . |
| H181 | . | . | . | . | . | . | . | . | . | . | . | . | G | C | . | . | . | . | T | . | . | . | . | . | . | . | . | T | T | . | . | . | . | . | . | . | . | C | . | G | . | . |
| H182 | . | . | . | . | . | . | . | . | . | . | . | . | G | C | . | . | . | . | . | . | . | C | . | . | . | . | . | T | T | . | . | . | . | . | . | . | . | . | . | . | . | . |
| H183 | . | . | . | . | . | . | . | . | . | . | . | . | G | . | . | . | . | . | . | . | . | . | . | . | . | A | . | T | T | . | . | . | . | . | . | . | . | . | . | . | . | . |
| H184 | . | . | . | . | . | . | . | . | . | . | . | . | G | . | . | . | . | . | . | A | . | . | . | . | . | . | . | . | . | . | . | . | . | . | . | T | . | C | . | . | . | . |
| H185 | . | . | . | . | . | . | . | . | . | . | . | . | G | . | . | . | . | . | . | A | C | . | . | . | . | . | . | . | . | . | . | . | . | . | . | T | G | | . | G | . | C |
| H186 | . | . | . | . | . | . | . | . | . | . | . | . | G | . | . | . | . | . | . | . | C | C | . | . | . | . | . | T | T | . | . | . | . | . | . | . | . | C | . | . | . | C |
| H187 | . | . | . | . | . | . | . | . | . | . | . | . | G | . | . | . | . | . | . | . | C | C | . | . | . | . | . | . | . | . | . | . | . | . | . | . | . | . | . | . | . | C |
| H188 | . | . | . | . | . | . | . | . | . | . | . | . | G | . | . | . | . | . | . | . | C | C | . | . | . | . | . | T | T | . | . | . | . | . | . | . | . | . | . | . | . | . |
| H189 | . | . | . | . | . | . | . | . | . | . | . | . | G | . | . | . | . | . | . | . | C | C | . | . | . | . | . | T | T | . | . | . | . | . | . | . | . | . | . | G | . | . |
| H190 | . | . | . | . | . | . | . | . | . | . | . | . | G | . | . | . | . | . | . | . | . | C | . | . | . | . | . | T | T | . | . | . | . | . | . | T | . | . | . | G | . | C |
| H191 | . | . | . | . | . | . | . | . | . | . | . | . | G | . | . | . | . | . | . | . | . | C | . | . | . | . | . | . | T | . | . | . | . | . | . | . | . | . | . | G | . | . |
| H192 | . | . | . | . | . | . | . | . | . | . | . | . | G | . | . | . | . | . | . | . | . | C | . | . | . | . | . | . | T | . | . | . | . | . | . | . | . | . | . | . | . | C |
| H193 | . | . | . | . | . | . | . | . | . | . | . | . | G | . | . | . | . | . | . | . | . | C | . | . | . | . | . | T | T | . | . | . | . | . | . | . | . | C | . | . | . | . |
| H194 | . | . | . | . | . | . | . | . | . | . | . | . | G | . | . | . | . | . | . | . | . | . | A | . | . | A | . | T | T | . | . | . | . | . | . | . | . | . | . | G | . | . |
| H195 | . | . | . | . | . | . | . | . | . | . | . | . | G | . | . | . | . | . | . | . | . | . | . | . | . | . | . | . | T | . | . | . | . | . | . | . | . | . | . | G | . | C |
| H196 | . | . | . | . | . | . | . | . | . | . | . | . | G | . | . | . | . | . | . | . | . | . | . | . | . | . | . | T | T | . | . | . | . | . | . | . | . | . | . | G | . | C |
| H197 | . | . | . | . | . | . | . | . | . | . | . | . | G | . | . | . | . | . | . | . | . | . | . | . | . | . | . | T | T | . | . | . | . | . | . | . | . | . | . | . | . | . |
| H198 | . | . | . | . | . | . | . | . | . | . | . | . | G | . | . | . | . | . | . | . | . | . | . | . | . | . | . | T | T | . | . | . | . | . | . | . | . | . | . | . | . | C |
| H199 | . | . | . | . | . | . | . | . | . | . | . | . | G | . | . | . | . | . | . | . | . | . | . | . | . | . | . | . | . | . | . | . | . | . | . | . | . | C | A | G | . | . |
| H200 | . | . | . | . | . | . | . | . | . | . | . | . | G | . | . | . | . | . | . | . | . | . | . | . | . | . | . | . | . | . | . | . | . | . | . | . | . | . | . | . | . | . |
| H201 | . | . | . | . | . | . | . | . | . | . | . | . | G | . | . | . | . | . | . | . | . | . | . | . | . | . | . | . | T | . | . | . | . | . | . | T | . | . | . | G | . | C |
| H202 | . | . | . | . | . | . | . | . | . | . | . | . | G | . | . | . | . | . | . | . | . | . | . | . | . | . | . | . | T | . | . | . | . | . | . | | . | . | A | G | . | C |
| H203 | . | . | . | . | . | . | . | . | . | . | . | . | G | . | . | . | . | . | . | . | . | . | . | . | . | A | . | . | T | . | . | . | . | . | . | | . | . | . | . | . | . |
| H204 | . | . | . | . | . | . | . | . | . | . | . | . | G | . | . | . | . | . | . | . | . | . | . | . | . | A | . | . | T | . | . | . | . | . | . | | . | . | . | G | . | C |
| H205 | . | . | . | . | . | . | . | . | . | . | . | . | G | . | . | . | . | . | . | . | . | . | . | . | . | A | . | T | T | . | . | . | . | . | . | T | . | . | . | G | . | . |
| H206 | . | . | . | . | . | . | . | . | . | . | . | . | G | . | . | . | . | . | . | . | . | . | . | . | . | A | . | T | T | . | . | . | . | . | . | . | . | . | A | G | . | C |
| H207 | . | . | . | . | . | . | . | . | . | . | . | . | G | . | . | . | . | . | . | . | . | . | . | . | . | A | . | T | T | . | . | . | . | . | . | . | . | . | . | G | . | . |
| H208 | . | . | . | . | . | . | . | . | . | . | . | . | G | . | . | . | . | . | . | . | . | . | . | . | . | A | . | T | T | . | . | . | . | . | . | . | . | . | . | G | . | C |
| H209 | . | . | . | . | . | . | . | . | . | . | . | . | G | . | . | . | . | . | T | A | C | . | . | . | . | . | . | . | . | . | . | . | . | . | . | . | . | . | . | G | . | C |

| | Locus, Consensus, Character | | | | | | | | | | | | | | | | | | | | | | | | | | | | | | | | | | | | | | | | | |
|---|---|---|---|---|---|---|---|---|---|---|---|---|---|---|---|---|---|---|---|---|---|---|---|---|---|---|---|---|---|---|---|---|---|---|---|---|---|---|---|---|---|---|
| | **CDC** | | | | | | | | | | | | | **EF** | | | | | **HSP** | | | | | | | **MAP** | | | | **UBA** | | | | | | **UBC** | | | | | | |
| | **C** | **G** | **G** | **T** | **T** | **G** | **G** | **G** | **G** | **A** | **G** | **A** | **A** | **T** | **A** | **T** | **C** | **G** | **C** | **C** | **G** | **G** | **C** | **T** | **G** | **G** | **A** | **C** | **G** | **C** | **G** | **A** | **C** | **A** | **A** | **C** | **A** | **T** | **G** | **T** | **C** | **T** |
| | **i** | **n** | **i** | **i** | **i** | **i** | **n** | **n** | **i** | **n** | **i** | **n** | **i** | **i** | **n** | **n** | **i** | **i** | **i** | **i** | **i** | **i** | **i** | **i** | **i** | **i** | **n** | **i** | **i** | **i** | **i** | **i** | **i** | **i** | **i** | **i** | **i** | **i** | **i** | **i** | **i** | **i** |
| H210 | . | . | . | . | . | . | . | . | . | . | . | . | G | . | . | . | . | . | T | A | C | . | A | C | . | . | . | . | . | . | . | . | . | . | . | T | . | . | . | . | . | . |
| H211 | . | . | . | . | . | . | . | . | . | . | . | . | G | . | . | . | . | . | T | A | C | . | A | C | . | A | . | . | T | . | . | . | . | . | . | T | G | . | A | G | G | C |
| H212 | . | . | . | . | . | . | . | . | . | . | . | . | G | . | . | . | . | . | T | A | C | . | A | C | . | A | . | T | T | . | . | . | . | . | . | T | G | . | A | G | G | C |
| H213 | . | . | . | . | . | . | . | . | . | . | . | . | G | . | . | . | . | . | T | A | C | . | A | C | . | A | . | T | T | . | . | . | . | . | . | . | . | C | . | G | G | C |
| H214 | . | . | . | . | . | . | . | . | . | . | . | . | G | . | . | . | . | . | T | A | C | . | A | C | . | . | . | T | T | . | . | . | . | . | . | T | G | . | A | G | G | C |
| H215 | . | . | . | . | . | . | . | . | . | . | . | . | G | . | . | . | . | . | T | A | C | . | A | C | . | . | . | . | T | . | . | . | . | . | . | T | G | . | A | G | G | C |
| H216 | . | . | . | . | . | . | . | . | . | . | . | . | G | . | . | . | . | . | T | A | C | . | A | C | . | . | . | . | . | . | . | . | . | . | . | T | G | . | A | G | G | C |
| H217 | . | . | . | . | . | . | . | . | . | . | . | . | G | . | . | . | . | . | T | . | . | C | . | . | . | . | . | T | T | . | . | . | . | . | . | . | . | . | A | G | . | . |
| H218 | . | . | . | . | . | . | . | . | . | . | . | . | G | . | . | . | . | . | T | . | . | C | . | . | . | A | . | . | T | . | . | . | . | . | . | . | . | C | . | . | . | . |
| H219 | . | . | . | . | . | . | . | . | . | . | . | . | G | . | . | . | . | . | T | . | . | C | . | . | . | . | . | . | . | . | . | . | . | . | . | . | . | | . | . | . | . |
| H220 | . | . | . | . | . | . | . | . | . | . | . | . | G | . | . | . | . | . | T | . | . | . | . | . | . | A | . | . | T | . | . | . | . | . | . | . | . | | . | . | . | C |

Each locus comprises variable positions within a single nuclear gene; Most frequent or consensus nucleotide for each variable position: T, C, A, or G. Character type indicates whether each variable position is phylogenetically informative (i) or noninformative (n). To be informative, a position must have mutations that appear in at least two haplotypes.

**Table S3.** Number of haplotype and its distribution among different location.

| Sample | LJ | DH | BS | DL | QJ | ZT | YX | LC | WS | CX | KM | Total |
|---|---|---|---|---|---|---|---|---|---|---|---|---|
| H1 | 0 | 0 | 0 | 0 | 1 | 0 | 0 | 0 | 0 | 0 | 0 | 1 |
| H2 | 0 | 0 | 0 | 0 | 1 | 0 | 0 | 0 | 0 | 0 | 0 | 1 |
| H3 | 0 | 0 | 0 | 0 | 0 | 0 | 0 | 0 | 0 | 0 | 1 | 1 |
| H4 | 0 | 0 | 0 | 0 | 0 | 0 | 0 | 0 | 1 | 0 | 0 | 1 |
| H5 | 0 | 0 | 0 | 0 | 0 | 0 | 0 | 0 | 1 | 0 | 0 | 1 |
| H6 | 1 | 0 | 0 | 0 | 0 | 0 | 0 | 0 | 0 | 0 | 0 | 1 |
| H7 | 0 | 0 | 0 | 0 | 0 | 0 | 0 | 0 | 0 | 1 | 0 | 1 |
| H8 | 3 | 0 | 3 | 2 | 0 | 0 | 0 | 0 | 0 | 3 | 0 | 11 |
| H9 | 0 | 1 | 0 | 0 | 0 | 0 | 0 | 0 | 0 | 0 | 0 | 1 |
| H10 | 1 | 0 | 0 | 0 | 0 | 1 | 0 | 0 | 0 | 1 | 4 | 7 |
| H11 | 0 | 1 | 0 | 0 | 0 | 0 | 0 | 0 | 0 | 0 | 0 | 1 |
| H12 | 0 | 0 | 1 | 0 | 0 | 0 | 0 | 0 | 0 | 0 | 0 | 1 |
| H13 | 0 | 0 | 1 | 0 | 0 | 0 | 0 | 0 | 0 | 0 | 0 | 1 |
| H14 | 0 | 0 | 0 | 0 | 0 | 0 | 0 | 0 | 1 | 0 | 0 | 1 |
| H15 | 0 | 0 | 1 | 2 | 0 | 0 | 1 | 5 | 0 | 0 | 1 | 10 |
| H16 | 0 | 0 | 0 | 0 | 0 | 1 | 0 | 0 | 0 | 0 | 0 | 1 |
| H17 | 0 | 0 | 0 | 0 | 0 | 0 | 1 | 0 | 0 | 0 | 0 | 1 |
| H18 | 0 | 1 | 0 | 0 | 0 | 1 | 6 | 2 | 1 | 0 | 2 | 13 |
| H19 | 0 | 0 | 0 | 0 | 0 | 2 | 0 | 0 | 0 | 0 | 0 | 2 |
| H20 | 0 | 0 | 0 | 0 | 0 | 0 | 4 | 2 | 1 | 0 | 2 | 9 |
| H21 | 0 | 0 | 0 | 0 | 1 | 0 | 0 | 0 | 0 | 0 | 0 | 1 |
| H22 | 0 | 0 | 0 | 1 | 0 | 0 | 0 | 0 | 0 | 0 | 0 | 1 |
| H23 | 0 | 0 | 1 | 0 | 7 | 0 | 0 | 0 | 2 | 0 | 0 | 10 |
| H24 | 0 | 0 | 0 | 0 | 1 | 0 | 0 | 0 | 0 | 0 | 0 | 1 |
| H25 | 0 | 0 | 1 | 0 | 0 | 0 | 0 | 0 | 0 | 0 | 0 | 1 |
| H26 | 0 | 0 | 1 | 0 | 0 | 0 | 0 | 0 | 0 | 0 | 0 | 1 |
| H27 | 0 | 0 | 0 | 0 | 0 | 0 | 1 | 0 | 0 | 0 | 0 | 1 |
| H28 | 0 | 0 | 0 | 1 | 0 | 0 | 0 | 0 | 0 | 0 | 0 | 1 |
| H29 | 0 | 1 | 0 | 0 | 0 | 0 | 0 | 0 | 0 | 0 | 0 | 1 |
| H30 | 0 | 0 | 1 | 0 | 0 | 0 | 0 | 0 | 0 | 0 | 0 | 1 |
| H31 | 1 | 0 | 0 | 0 | 0 | 0 | 0 | 0 | 0 | 0 | 0 | 1 |
| H32 | 1 | 0 | 0 | 0 | 0 | 0 | 0 | 0 | 0 | 0 | 0 | 1 |
| H33 | 0 | 1 | 0 | 0 | 0 | 0 | 0 | 0 | 0 | 0 | 0 | 1 |
| H34 | 1 | 0 | 0 | 0 | 0 | 0 | 0 | 0 | 0 | 0 | 0 | 1 |
| H35 | 0 | 0 | 1 | 0 | 0 | 0 | 0 | 0 | 0 | 0 | 0 | 1 |
| H36 | 1 | 0 | 0 | 0 | 0 | 0 | 0 | 0 | 0 | 1 | 0 | 2 |
| H37 | 1 | 0 | 0 | 0 | 0 | 0 | 0 | 0 | 0 | 0 | 0 | 1 |
| H38 | 0 | 0 | 0 | 0 | 0 | 0 | 0 | 0 | 0 | 0 | 1 | 1 |
| H39 | 0 | 2 | 0 | 0 | 0 | 0 | 0 | 0 | 0 | 0 | 1 | 3 |
| H40 | 0 | 0 | 1 | 0 | 0 | 0 | 0 | 0 | 0 | 0 | 0 | 1 |
| H41 | 0 | 1 | 0 | 0 | 0 | 0 | 0 | 0 | 0 | 0 | 0 | 1 |
| H42 | 0 | 0 | 0 | 0 | 0 | 0 | 0 | 0 | 1 | 0 | 0 | 1 |
| H43 | 0 | 0 | 0 | 0 | 0 | 0 | 0 | 1 | 0 | 0 | 0 | 1 |
| H44 | 0 | 1 | 0 | 0 | 0 | 0 | 0 | 0 | 0 | 0 | 0 | 1 |
| H45 | 0 | 0 | 1 | 0 | 0 | 0 | 0 | 0 | 0 | 0 | 0 | 1 |
| H46 | 0 | 1 | 0 | 0 | 0 | 0 | 0 | 0 | 0 | 0 | 0 | 1 |
| H47 | 0 | 0 | 0 | 0 | 0 | 1 | 0 | 0 | 0 | 0 | 0 | 1 |
| H48 | 0 | 0 | 1 | 0 | 0 | 0 | 0 | 0 | 0 | 0 | 0 | 1 |
| H49 | 0 | 1 | 0 | 0 | 0 | 0 | 0 | 0 | 0 | 0 | 0 | 1 |

| Sample | LJ | DH | BS | DL | QJ | ZT | YX | LC | WS | CX | KM | Total |
|---|---|---|---|---|---|---|---|---|---|---|---|---|
| H50 | 0 | 0 | 0 | 1 | 0 | 0 | 0 | 0 | 0 | 0 | 0 | 1 |
| H51 | 0 | 0 | 1 | 1 | 0 | 0 | 0 | 0 | 0 | 0 | 0 | 2 |
| H52 | 0 | 0 | 0 | 1 | 0 | 0 | 0 | 0 | 0 | 0 | 0 | 1 |
| H53 | 0 | 0 | 0 | 1 | 0 | 0 | 0 | 0 | 0 | 0 | 0 | 1 |
| H54 | 0 | 2 | 0 | 0 | 0 | 0 | 1 | 0 | 0 | 0 | 0 | 3 |
| H55 | 0 | 1 | 0 | 0 | 0 | 0 | 0 | 0 | 0 | 0 | 0 | 1 |
| H56 | 0 | 1 | 0 | 0 | 0 | 0 | 0 | 0 | 0 | 0 | 0 | 1 |
| H57 | 0 | 1 | 0 | 0 | 0 | 0 | 0 | 0 | 0 | 0 | 0 | 1 |
| H58 | 0 | 1 | 0 | 0 | 0 | 0 | 0 | 0 | 0 | 0 | 0 | 1 |
| H59 | 1 | 0 | 0 | 0 | 0 | 1 | 0 | 0 | 0 | 0 | 0 | 2 |
| H60 | 0 | 0 | 0 | 0 | 1 | 0 | 0 | 0 | 0 | 0 | 0 | 1 |
| H61 | 0 | 0 | 0 | 0 | 1 | 0 | 0 | 0 | 0 | 0 | 0 | 1 |
| H62 | 0 | 0 | 0 | 0 | 0 | 1 | 0 | 0 | 0 | 0 | 0 | 1 |
| H63 | 0 | 0 | 0 | 1 | 0 | 0 | 0 | 0 | 0 | 0 | 0 | 1 |
| H64 | 0 | 1 | 0 | 0 | 0 | 1 | 0 | 0 | 0 | 0 | 0 | 2 |
| H65 | 1 | 0 | 0 | 0 | 0 | 1 | 0 | 0 | 0 | 0 | 0 | 2 |
| H66 | 0 | 0 | 0 | 1 | 0 | 0 | 0 | 0 | 0 | 0 | 0 | 1 |
| H67 | 1 | 0 | 0 | 0 | 0 | 0 | 0 | 0 | 0 | 0 | 0 | 1 |
| H68 | 1 | 1 | 0 | 1 | 0 | 0 | 0 | 0 | 0 | 0 | 2 | 5 |
| H69 | 1 | 0 | 0 | 0 | 0 | 0 | 0 | 0 | 0 | 0 | 0 | 1 |
| H70 | 0 | 0 | 0 | 0 | 1 | 0 | 0 | 0 | 0 | 0 | 0 | 1 |
| H71 | 0 | 0 | 0 | 0 | 0 | 0 | 0 | 0 | 0 | 1 | 0 | 1 |
| H72 | 3 | 0 | 0 | 0 | 0 | 2 | 0 | 0 | 0 | 0 | 0 | 5 |
| H73 | 0 | 0 | 0 | 0 | 0 | 1 | 0 | 0 | 0 | 0 | 0 | 1 |
| H74 | 0 | 0 | 0 | 1 | 0 | 0 | 0 | 0 | 0 | 0 | 0 | 1 |
| H75 | 0 | 0 | 0 | 1 | 0 | 0 | 0 | 0 | 0 | 0 | 0 | 1 |
| H76 | 2 | 0 | 1 | 1 | 0 | 0 | 0 | 0 | 0 | 1 | 0 | 5 |
| H77 | 0 | 0 | 0 | 0 | 0 | 0 | 0 | 1 | 0 | 0 | 0 | 1 |
| H78 | 1 | 0 | 0 | 0 | 0 | 0 | 0 | 1 | 1 | 0 | 0 | 3 |
| H79 | 1 | 0 | 0 | 0 | 0 | 0 | 0 | 0 | 0 | 0 | 2 | 3 |
| H80 | 0 | 1 | 0 | 0 | 0 | 0 | 0 | 0 | 0 | 0 | 0 | 1 |
| H81 | 0 | 0 | 0 | 0 | 0 | 0 | 0 | 0 | 0 | 0 | 1 | 1 |
| H82 | 0 | 0 | 1 | 0 | 0 | 0 | 0 | 0 | 0 | 2 | 0 | 3 |
| H83 | 1 | 0 | 1 | 0 | 0 | 0 | 0 | 0 | 0 | 0 | 1 | 3 |
| H84 | 0 | 0 | 0 | 1 | 0 | 0 | 1 | 0 | 0 | 0 | 0 | 2 |
| H85 | 0 | 0 | 0 | 0 | 0 | 0 | 0 | 0 | 0 | 0 | 1 | 1 |
| H86 | 3 | 4 | 2 | 1 | 0 | 0 | 0 | 0 | 0 | 1 | 3 | 14 |
| H87 | 0 | 0 | 0 | 3 | 0 | 0 | 0 | 0 | 0 | 0 | 0 | 3 |
| H88 | 0 | 0 | 0 | 1 | 0 | 0 | 0 | 0 | 0 | 0 | 0 | 1 |
| H89 | 1 | 0 | 0 | 0 | 0 | 0 | 0 | 0 | 0 | 0 | 0 | 1 |
| H90 | 1 | 0 | 0 | 0 | 0 | 0 | 0 | 0 | 0 | 0 | 0 | 1 |
| H91 | 1 | 0 | 0 | 0 | 0 | 0 | 0 | 0 | 0 | 0 | 0 | 1 |
| H92 | 0 | 0 | 0 | 1 | 0 | 0 | 0 | 0 | 0 | 0 | 0 | 1 |
| H93 | 0 | 0 | 0 | 0 | 0 | 0 | 0 | 0 | 0 | 0 | 1 | 1 |
| H94 | 1 | 0 | 2 | 1 | 0 | 0 | 0 | 0 | 0 | 0 | 0 | 4 |
| H95 | 0 | 0 | 0 | 1 | 0 | 0 | 0 | 0 | 0 | 0 | 0 | 1 |
| H96 | 0 | 1 | 0 | 0 | 0 | 0 | 0 | 0 | 0 | 0 | 0 | 1 |
| H97 | 0 | 0 | 0 | 1 | 0 | 0 | 0 | 0 | 0 | 0 | 0 | 1 |
| H98 | 0 | 0 | 0 | 1 | 0 | 0 | 0 | 0 | 0 | 0 | 0 | 1 |
| H99 | 0 | 0 | 0 | 0 | 0 | 0 | 0 | 0 | 0 | 0 | 1 | 1 |
| H100 | 0 | 1 | 0 | 0 | 0 | 0 | 0 | 0 | 0 | 0 | 0 | 1 |
| H101 | 0 | 1 | 0 | 0 | 0 | 0 | 0 | 0 | 0 | 0 | 0 | 1 |

| Sample | LJ | DH | BS | DL | QJ | ZT | YX | LC | WS | CX | KM | Total |
|---|---|---|---|---|---|---|---|---|---|---|---|---|
| H102 | 0 | 0 | 0 | 0 | 0 | 1 | 0 | 0 | 0 | 0 | 0 | 1 |
| H103 | 1 | 0 | 0 | 0 | 0 | 0 | 0 | 0 | 0 | 0 | 0 | 1 |
| H104 | 0 | 0 | 0 | 2 | 0 | 0 | 1 | 0 | 0 | 0 | 0 | 3 |
| H105 | 0 | 0 | 0 | 1 | 0 | 0 | 0 | 0 | 0 | 0 | 0 | 1 |
| H106 | 0 | 0 | 0 | 0 | 0 | 0 | 1 | 0 | 0 | 0 | 0 | 1 |
| H107 | 0 | 0 | 0 | 0 | 0 | 1 | 0 | 0 | 1 | 0 | 0 | 2 |
| H108 | 0 | 1 | 0 | 0 | 0 | 0 | 0 | 0 | 0 | 0 | 0 | 1 |
| H109 | 0 | 0 | 0 | 0 | 0 | 0 | 1 | 1 | 0 | 0 | 0 | 2 |
| H110 | 0 | 0 | 0 | 0 | 1 | 0 | 0 | 0 | 0 | 0 | 0 | 1 |
| H111 | 0 | 0 | 0 | 0 | 0 | 0 | 0 | 0 | 0 | 1 | 0 | 1 |
| H112 | 0 | 0 | 0 | 0 | 0 | 0 | 0 | 0 | 0 | 1 | 0 | 1 |
| H113 | 0 | 0 | 0 | 0 | 0 | 0 | 0 | 1 | 0 | 0 | 0 | 1 |
| H114 | 0 | 0 | 0 | 0 | 1 | 0 | 0 | 0 | 1 | 0 | 0 | 2 |
| H115 | 0 | 0 | 0 | 0 | 1 | 0 | 0 | 1 | 0 | 0 | 0 | 2 |
| H116 | 0 | 0 | 0 | 0 | 0 | 1 | 0 | 1 | 0 | 0 | 0 | 2 |
| H117 | 0 | 0 | 0 | 0 | 1 | 0 | 0 | 1 | 0 | 0 | 0 | 2 |
| H118 | 0 | 0 | 0 | 0 | 0 | 1 | 0 | 0 | 0 | 0 | 0 | 1 |
| H119 | 0 | 0 | 0 | 0 | 0 | 0 | 0 | 0 | 0 | 1 | 0 | 1 |
| H120 | 0 | 0 | 0 | 0 | 0 | 0 | 0 | 1 | 0 | 0 | 0 | 1 |
| H121 | 0 | 0 | 0 | 0 | 0 | 0 | 0 | 0 | 1 | 0 | 0 | 1 |
| H122 | 0 | 0 | 0 | 0 | 0 | 0 | 0 | 0 | 0 | 1 | 0 | 1 |
| H123 | 0 | 0 | 0 | 0 | 1 | 0 | 0 | 0 | 0 | 0 | 0 | 1 |
| H124 | 0 | 0 | 0 | 0 | 0 | 0 | 0 | 0 | 0 | 1 | 0 | 1 |
| H125 | 0 | 0 | 0 | 0 | 0 | 0 | 0 | 1 | 0 | 0 | 0 | 1 |
| H126 | 0 | 0 | 0 | 0 | 0 | 1 | 0 | 0 | 0 | 0 | 0 | 1 |
| H127 | 0 | 1 | 0 | 0 | 0 | 0 | 0 | 0 | 0 | 0 | 0 | 1 |
| H128 | 0 | 0 | 0 | 0 | 0 | 0 | 0 | 0 | 0 | 1 | 0 | 1 |
| H129 | 0 | 0 | 0 | 0 | 0 | 1 | 0 | 0 | 0 | 0 | 0 | 1 |
| H130 | 0 | 0 | 0 | 0 | 0 | 0 | 1 | 0 | 0 | 0 | 0 | 1 |
| H131 | 0 | 0 | 0 | 0 | 0 | 0 | 0 | 0 | 0 | 0 | 1 | 1 |
| H132 | 0 | 0 | 0 | 0 | 0 | 0 | 0 | 0 | 0 | 0 | 1 | 1 |
| H133 | 0 | 0 | 0 | 0 | 0 | 0 | 0 | 0 | 0 | 0 | 1 | 1 |
| H134 | 0 | 0 | 0 | 0 | 0 | 1 | 0 | 0 | 0 | 0 | 0 | 1 |
| H135 | 0 | 0 | 0 | 1 | 0 | 0 | 0 | 0 | 0 | 0 | 0 | 1 |
| H136 | 0 | 0 | 0 | 0 | 0 | 1 | 0 | 0 | 0 | 0 | 0 | 1 |
| H137 | 0 | 0 | 0 | 0 | 0 | 0 | 0 | 0 | 0 | 0 | 1 | 1 |
| H138 | 0 | 0 | 0 | 0 | 0 | 0 | 0 | 0 | 0 | 0 | 1 | 1 |
| H139 | 0 | 0 | 0 | 0 | 0 | 0 | 0 | 0 | 1 | 0 | 0 | 1 |
| H140 | 0 | 0 | 0 | 0 | 0 | 1 | 1 | 0 | 0 | 0 | 0 | 2 |
| H141 | 0 | 0 | 0 | 0 | 0 | 0 | 0 | 0 | 2 | 0 | 0 | 2 |
| H142 | 0 | 0 | 0 | 0 | 0 | 0 | 0 | 0 | 1 | 0 | 0 | 1 |
| H143 | 0 | 0 | 0 | 0 | 0 | 0 | 0 | 0 | 0 | 0 | 1 | 1 |
| H144 | 0 | 0 | 0 | 0 | 0 | 2 | 0 | 0 | 0 | 0 | 0 | 2 |
| H145 | 0 | 0 | 0 | 0 | 0 | 1 | 0 | 0 | 0 | 0 | 0 | 1 |
| H146 | 0 | 0 | 0 | 0 | 0 | 0 | 0 | 0 | 1 | 0 | 0 | 1 |
| H147 | 0 | 0 | 0 | 0 | 0 | 0 | 1 | 0 | 0 | 0 | 0 | 1 |
| H148 | 0 | 0 | 0 | 0 | 0 | 0 | 1 | 0 | 0 | 0 | 0 | 1 |
| H149 | 0 | 0 | 0 | 0 | 0 | 0 | 0 | 0 | 0 | 0 | 2 | 2 |
| H150 | 0 | 0 | 0 | 0 | 0 | 0 | 0 | 0 | 0 | 0 | 1 | 1 |
| H151 | 0 | 0 | 0 | 1 | 0 | 0 | 0 | 0 | 0 | 0 | 0 | 1 |
| H152 | 0 | 0 | 0 | 0 | 0 | 0 | 0 | 0 | 0 | 1 | 0 | 1 |
| H153 | 0 | 0 | 0 | 0 | 0 | 0 | 1 | 0 | 0 | 0 | 0 | 1 |

| Sample | LJ | DH | BS | DL | QJ | ZT | YX | LC | WS | CX | KM | Total |
|---|---|---|---|---|---|---|---|---|---|---|---|---|
| H154 | 0 | 0 | 0 | 0 | 0 | 0 | 0 | 0 | 0 | 1 | 0 | 1 |
| H155 | 0 | 0 | 0 | 0 | 0 | 1 | 0 | 0 | 1 | 0 | 0 | 2 |
| H156 | 0 | 0 | 0 | 0 | 0 | 0 | 0 | 0 | 0 | 1 | 0 | 1 |
| H157 | 0 | 0 | 1 | 0 | 0 | 0 | 0 | 0 | 0 | 0 | 0 | 1 |
| H158 | 0 | 0 | 0 | 0 | 1 | 0 | 0 | 0 | 0 | 0 | 1 | 2 |
| H159 | 0 | 0 | 0 | 0 | 0 | 1 | 0 | 0 | 0 | 0 | 0 | 1 |
| H160 | 0 | 0 | 0 | 0 | 0 | 1 | 0 | 0 | 0 | 0 | 0 | 1 |
| H161 | 0 | 0 | 0 | 0 | 0 | 0 | 0 | 0 | 0 | 0 | 1 | 1 |
| H162 | 0 | 0 | 0 | 2 | 0 | 0 | 0 | 0 | 0 | 0 | 0 | 2 |
| H163 | 0 | 0 | 0 | 0 | 1 | 0 | 0 | 0 | 0 | 0 | 0 | 1 |
| H164 | 0 | 0 | 0 | 1 | 0 | 1 | 0 | 0 | 0 | 0 | 0 | 2 |
| H165 | 0 | 0 | 0 | 0 | 1 | 0 | 0 | 0 | 0 | 0 | 0 | 1 |
| H166 | 0 | 0 | 0 | 0 | 0 | 0 | 0 | 0 | 0 | 0 | 1 | 1 |
| H167 | 0 | 0 | 0 | 0 | 0 | 0 | 0 | 0 | 1 | 0 | 0 | 1 |
| H168 | 0 | 0 | 0 | 0 | 0 | 0 | 0 | 0 | 0 | 0 | 1 | 1 |
| H169 | 0 | 0 | 0 | 0 | 0 | 0 | 0 | 0 | 0 | 1 | 0 | 1 |
| H170 | 0 | 0 | 0 | 0 | 0 | 0 | 0 | 0 | 0 | 0 | 1 | 1 |
| H171 | 0 | 0 | 0 | 0 | 0 | 0 | 1 | 0 | 0 | 0 | 0 | 1 |
| H172 | 0 | 0 | 0 | 0 | 0 | 0 | 0 | 0 | 0 | 0 | 1 | 1 |
| H173 | 0 | 0 | 0 | 0 | 0 | 0 | 0 | 0 | 1 | 0 | 2 | 3 |
| H174 | 0 | 0 | 0 | 0 | 0 | 0 | 0 | 0 | 0 | 0 | 1 | 1 |
| H175 | 0 | 0 | 0 | 0 | 0 | 0 | 0 | 0 | 0 | 0 | 1 | 1 |
| H176 | 0 | 0 | 0 | 0 | 0 | 0 | 0 | 0 | 2 | 0 | 0 | 2 |
| H177 | 0 | 0 | 0 | 0 | 0 | 0 | 0 | 0 | 0 | 0 | 1 | 1 |
| H178 | 0 | 0 | 0 | 0 | 0 | 2 | 0 | 0 | 0 | 0 | 0 | 2 |
| H179 | 0 | 0 | 0 | 0 | 0 | 0 | 0 | 0 | 0 | 1 | 0 | 1 |
| H180 | 0 | 0 | 0 | 0 | 0 | 0 | 0 | 0 | 0 | 1 | 0 | 1 |
| H181 | 0 | 0 | 0 | 0 | 0 | 0 | 1 | 0 | 0 | 0 | 0 | 1 |
| H182 | 0 | 0 | 0 | 0 | 0 | 0 | 1 | 0 | 0 | 0 | 0 | 1 |
| H183 | 0 | 0 | 0 | 0 | 0 | 0 | 1 | 0 | 1 | 0 | 0 | 2 |
| H184 | 0 | 0 | 0 | 1 | 0 | 0 | 0 | 0 | 0 | 0 | 0 | 1 |
| H185 | 0 | 0 | 0 | 0 | 1 | 0 | 0 | 0 | 0 | 0 | 0 | 1 |
| H186 | 0 | 0 | 0 | 0 | 0 | 0 | 0 | 0 | 0 | 0 | 1 | 1 |
| H187 | 0 | 0 | 0 | 0 | 0 | 0 | 0 | 0 | 0 | 0 | 1 | 1 |
| H188 | 0 | 0 | 0 | 0 | 0 | 0 | 0 | 0 | 0 | 0 | 1 | 1 |
| H189 | 0 | 0 | 0 | 0 | 0 | 1 | 0 | 0 | 0 | 0 | 0 | 1 |
| H190 | 0 | 0 | 0 | 0 | 0 | 0 | 1 | 0 | 0 | 0 | 0 | 1 |
| H191 | 0 | 0 | 0 | 0 | 0 | 0 | 1 | 0 | 0 | 0 | 0 | 1 |
| H192 | 0 | 0 | 0 | 0 | 0 | 0 | 0 | 1 | 0 | 0 | 0 | 1 |
| H193 | 0 | 0 | 0 | 0 | 0 | 0 | 1 | 0 | 0 | 0 | 0 | 1 |
| H194 | 0 | 0 | 0 | 0 | 0 | 0 | 0 | 1 | 0 | 0 | 0 | 1 |
| H195 | 0 | 0 | 0 | 0 | 1 | 0 | 0 | 1 | 0 | 0 | 0 | 2 |
| H196 | 0 | 0 | 0 | 0 | 1 | 0 | 0 | 0 | 0 | 0 | 0 | 1 |
| H197 | 0 | 0 | 0 | 0 | 0 | 0 | 1 | 1 | 0 | 0 | 0 | 2 |
| H198 | 0 | 0 | 0 | 0 | 0 | 0 | 1 | 0 | 0 | 0 | 0 | 1 |
| H199 | 0 | 0 | 0 | 0 | 1 | 0 | 0 | 0 | 0 | 0 | 0 | 1 |
| H200 | 0 | 0 | 0 | 0 | 0 | 0 | 1 | 0 | 0 | 0 | 0 | 1 |
| H201 | 0 | 0 | 0 | 0 | 0 | 0 | 1 | 0 | 0 | 0 | 0 | 1 |
| H202 | 0 | 0 | 0 | 0 | 0 | 0 | 0 | 1 | 0 | 0 | 0 | 1 |
| H203 | 0 | 0 | 0 | 0 | 0 | 0 | 1 | 0 | 0 | 0 | 0 | 1 |
| H204 | 0 | 0 | 0 | 0 | 0 | 0 | 1 | 0 | 0 | 0 | 0 | 1 |
| H205 | 0 | 0 | 0 | 0 | 0 | 0 | 0 | 1 | 0 | 0 | 0 | 1 |

| Sample | LJ | DH | BS | DL | QJ | ZT | YX | LC | WS | CX | KM | Total |
|---|---|---|---|---|---|---|---|---|---|---|---|---|
| H206 | 0 | 0 | 0 | 0 | 0 | 0 | 0 | 1 | 0 | 0 | 0 | 1 |
| H207 | 0 | 0 | 0 | 0 | 0 | 0 | 1 | 0 | 0 | 0 | 0 | 1 |
| H208 | 0 | 0 | 0 | 0 | 0 | 0 | 0 | 1 | 0 | 0 | 0 | 1 |
| H209 | 0 | 0 | 0 | 0 | 1 | 0 | 0 | 0 | 0 | 0 | 0 | 1 |
| H210 | 1 | 0 | 0 | 0 | 0 | 0 | 0 | 0 | 0 | 0 | 0 | 1 |
| H211 | 0 | 0 | 0 | 0 | 0 | 0 | 0 | 0 | 1 | 0 | 0 | 1 |
| H212 | 0 | 0 | 0 | 0 | 0 | 0 | 0 | 0 | 1 | 0 | 0 | 1 |
| H213 | 0 | 0 | 0 | 0 | 0 | 0 | 0 | 0 | 0 | 1 | 0 | 1 |
| H214 | 0 | 0 | 0 | 0 | 0 | 1 | 0 | 0 | 0 | 2 | 0 | 3 |
| H215 | 0 | 0 | 0 | 0 | 1 | 0 | 0 | 0 | 0 | 0 | 0 | 1 |
| H216 | 0 | 0 | 0 | 0 | 4 | 1 | 0 | 0 | 0 | 0 | 0 | 5 |
| H217 | 0 | 0 | 0 | 0 | 0 | 0 | 0 | 0 | 0 | 0 | 1 | 1 |
| H218 | 0 | 0 | 0 | 0 | 0 | 0 | 0 | 1 | 0 | 0 | 0 | 1 |
| H219 | 0 | 0 | 0 | 0 | 0 | 0 | 1 | 0 | 0 | 0 | 0 | 1 |
| H220 | 0 | 0 | 0 | 0 | 0 | 0 | 1 | 0 | 0 | 0 | 0 | 1 |
| SNP locus | 26 | 34 | 18 | 27 | 24 | 26 | 26 | 23 | 33 | 26 | 25 | 42 |
| Haplotype | 25 | 24 | 19 | 30 | 23 | 30 | 31 | 22 | 22 | 22 | 37 | 220 |
| P.hap | 13 | 18 | 10 | 20 | 16 | 17 | 21 | 12 | 13 | 15 | 26 | |
| Samples | 32 | 29 | 23 | 36 | 32 | 34 | 39 | 28 | 25 | 26 | 48 | 352 |

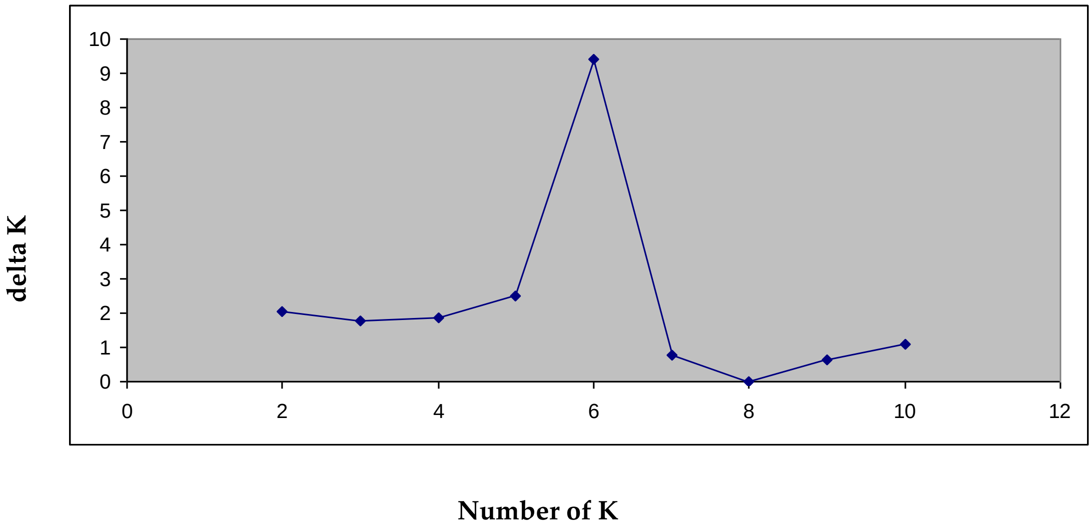


**Figure S1.** The best number of group among location estimated by Evano test methods.

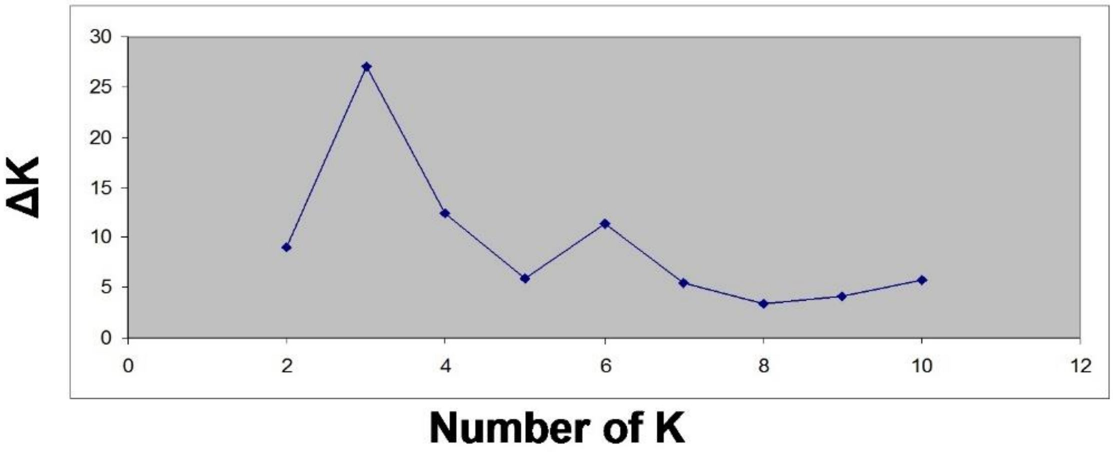


**Figure S2.** The determination of best number cluster among 220 haplotypes by Evano test methods.

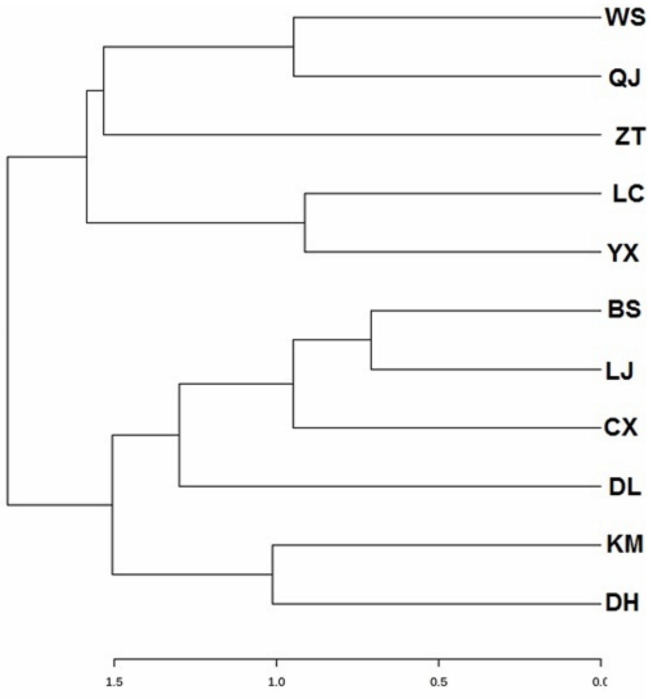


**Figure S3.** Six distinct groups found among 11 locations.
